# Deconstructing the Interoperability Stack: A Comparative Technical Analysis of HL7 v2, CDA, and the FHIR Resource Framework

**DOI:** 10.34133/csbj.0168

**Published:** 2026-08-05

**Authors:** Vibha Tiwari, Shreecha Jha, Rebakah Geddam, Muhammad Awais, Muhammad Ahmed Khan, Hemant Ghayvat

**Affiliations:** ^1^ Centre for Artificial Intelligence, Madhav Institute of Technology and Science, Gwalior, India.; ^2^ Department of Computer Science and Media Technology, eHealth Institute, Linnaeus University, Växjö, Sweden.; ^3^ Unitedworld Institute of Technology, Karnavati University, Gujarat, India.; ^4^ Memorial Sloan Kettering Cancer Center, New York, NY, USA.; ^5^ KITE Research Center, Toronto Rehabilitation Institute, University Health Network (UHN), 550 University Avenue, Toronto, ON, Canada.; ^6^ IMT, Department of Humanities and Technology, Roskilde University, Roskilde, Denmark.

## Abstract

This structured scoping review aims to provide a technical comparison of the development of interoperability standards in healthcare, from the early electronic health record systems, HL7 v2, HL7 v3/Clinical Document Architecture, to Fast Healthcare Interoperability Resources (FHIR). The review considers architectural foundations, semantic models, interoperability mechanisms, and conformance approaches of these standards in a consistent comparative context. With the introduction of the event-driven message paradigm (based on segments and trigger events), HL7 v2 became widely adopted in healthcare institutions; yet, a lot of customization at the local level often resulted in semantic incompleteness over the various environments within the healthcare industry. This led to the development of model-driven and document-centric semantic architectures such as HL7 v3 and Clinical Document Architecture to facilitate greater interoperability, but with implementation complexity and architectural rigidity, these were not widely embraced. FHIR then built on the experience of previous generations of interoperability, including the introduction of light-weight resource-oriented architectures, the RESTful way of interacting with resources, the profiling mechanism, and the introduction of a computable implementation guide that fits with web-native healthcare ecosystems. In contrast to the previous reviews, which concentrated mostly on individual interoperability standards or implementation areas, this review comparatively synthesizes technical and semantic development of interoperability architectures and investigates the treatment of any shortcomings found in preexisting architectures by the following standards. The synthesis indicates that FHIR currently represents one of the most scalable and implementation-oriented foundations for contemporary healthcare interoperability and future digital health integration.

## Graphical Abstract

**Figure:**
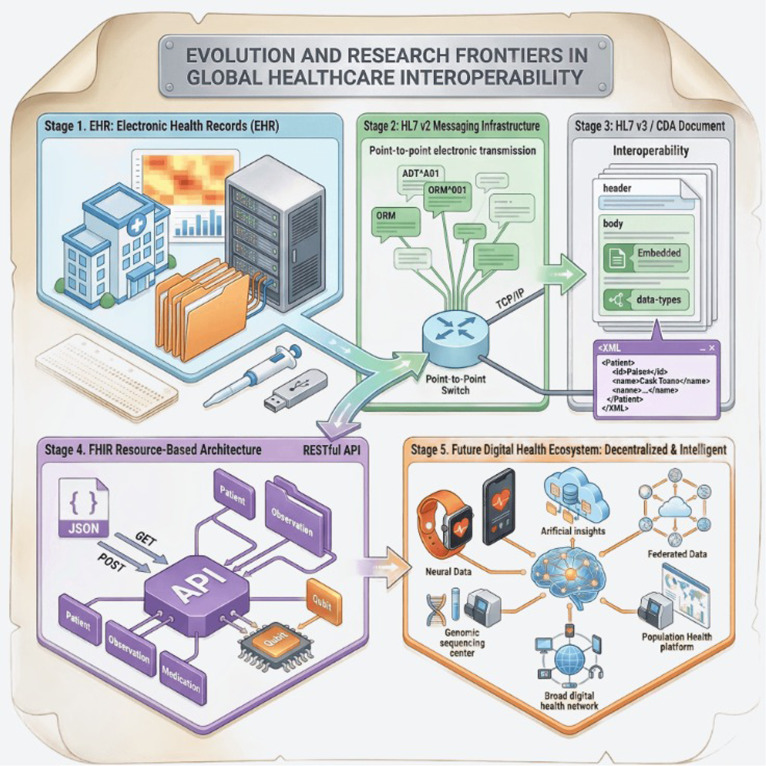

## Introduction

In today’s increasingly digital healthcare system, healthcare interoperability has become an essential need for successful clinical information exchange. While the massive use of electronic health records (EHRs) substantially contributed to the documentation of electronic records and to the management of clinical workflows, heterogeneous data structures, proprietary implementations and inconsistent semantic representations have still hindered the reliable interoperability between institutions and healthcare environments [1–6]. These difficulties spurred the creation of standard frameworks for interoperability designed to facilitate scalable, semantically consistent, and interoperable healthcare information exchange. In the course of this evolution, the HL7 family of standards increasingly influenced the development of current architectures for interoperability. To enable the wide institutional uptake of this standard, despite the ongoing and expected semantic variation, HL7 v2 added an event-driven messaging model with flexible implementation practices, optional segments, trigger-event based communication, and extensive local customization [7–9]. With this came the arrival of model-driven semantic structures in HL7 v3 and its Clinical Document Architecture (CDA), which was designed to increase semantic consistency and interoperability between different types of systems [9–11]. Later, the Fast Healthcare Interoperability Resources (FHIR) made strides in interoperability with an architecture that is lightweight, based on resources, and includes web-native features such as RESTful communication, modular integration, profiling, and application programming interface (API)-driven healthcare exchange [12–14].

This has been explored by a number of previous reviews, which have looked at interoperability standards, FHIR adoption, and health information exchange frameworks from implementation-oriented, regulatory, clinical, or application-specific perspectives [12,15–18]. However, limited attention has been devoted to a unified technical synthesis tracing the architectural, semantic, and interoperability evolution spanning EHR systems, HL7 v2, HL7 v3/CDA, and FHIR within a single longitudinal framework. Accordingly, this structured scoping review provides a comparative technical synthesis of the historical evolution, architectural foundations, semantic models, interoperability mechanisms, and conformance methodologies underlying these interoperability generations. The review also reviews the evolution of the standards that resolved the shortcomings of the previous ones while moving toward increasingly scalable, semantically consistent, and operationally flexible healthcare data exchange across different clinical settings, including heterogeneous ones [15–18].

## Methodology of the Structured Scoping Review

This structured scoping review provides a technical comparison and synthesis of the developments of healthcare interoperability standards, focusing on EHRs, HL7 v2, HL7 v3/CDA, and the architectural principles behind FHIR. The methodological framework was designed to support a transparent and reproducible synthesis of technical literature related to the historical evolution, semantic models, interoperability mechanisms, architectural structures, and implementation characteristics of these standards. The following research question was used to guide the review: What are the architectural and semantic changes in the evolution of healthcare interoperability standards from early EHR systems to HL7 v2, HL7 v3/CDA, and modern FHIR, and what were the shortcomings of previous interoperability standards that were addressed by each successive version?

To identify and screen relevant technical and biomedical literature, a structured scoping review approach was used, in accordance with the PRISMA Extension for Scoping Reviews (PRISMA-ScR) guidelines [19]. The review included basic research that has shaped the early development of EHR architectures, as well as recent research on HL7 v2, HL7 v3/CDA, and FHIR. Predefined inclusion and exclusion criteria were applied across major biomedical and technical databases through a comprehensive search strategy to retain studies addressing historical development, interoperability architectures, semantic representation models, implementation characteristics, and healthcare data exchange mechanisms [20]. Collectively, this methodological framework supported a longitudinal and technically informed synthesis of healthcare interoperability evolution. The final evidence base of the review is the result of the identification, deduplication, screening, selection, and comparative thematic synthesis procedures described in the following subsections.

### Research questions

The review was guided by the following research questions:

• How have healthcare interoperability standards evolved architecturally and semantically from early EHR systems through HL7 v2 and HL7 v3/CDA to modern FHIR?
• What structural, semantic, and interoperability limitations identified in preceding frameworks were addressed by successive generations of standards?
• How do messaging-based, document-centric, and resource-oriented interoperability paradigms differ with respect to semantic consistency, implementation flexibility, and interoperability scalability?

### Identification of records

**Fig. 1.**
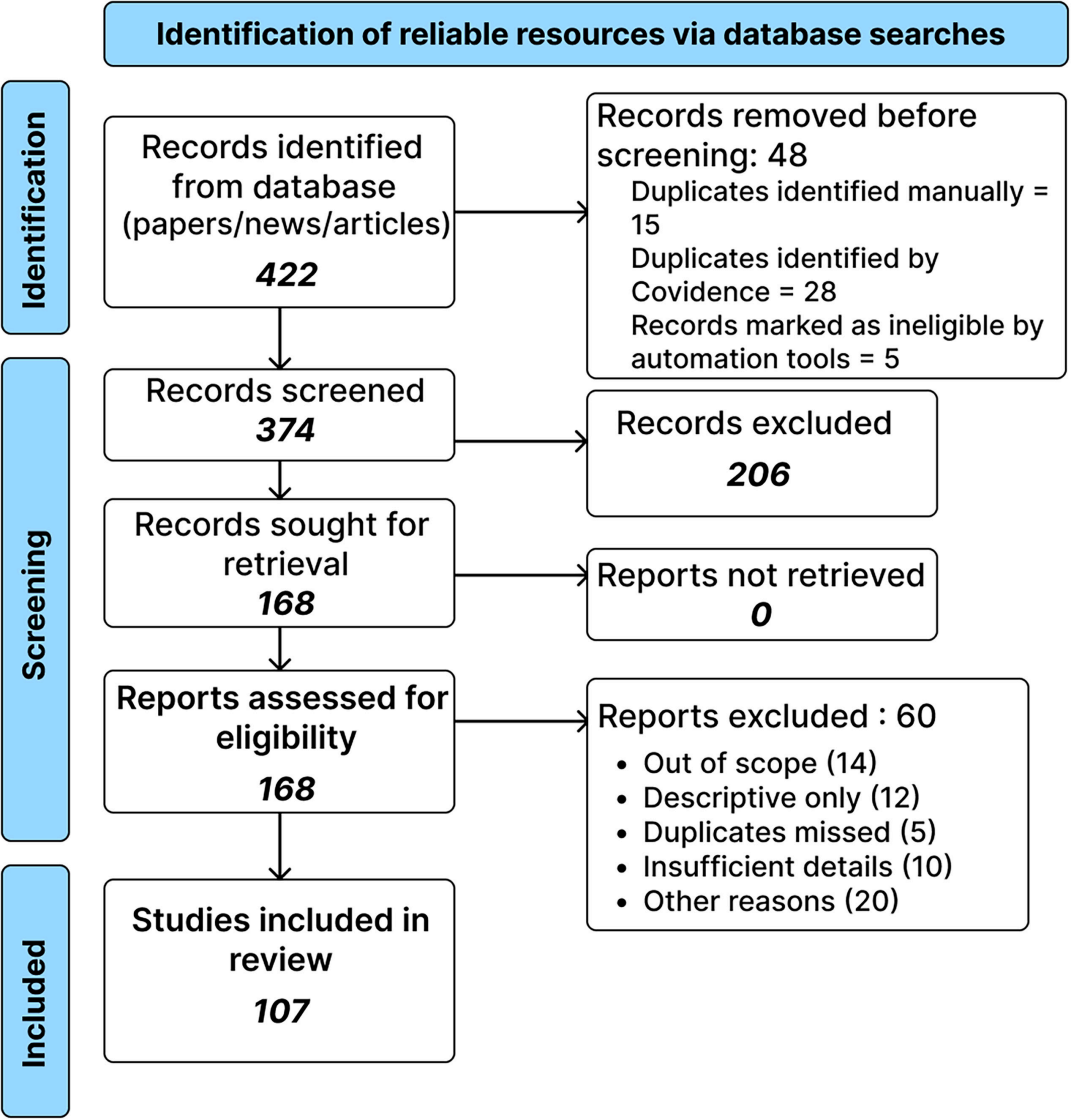
PRISMA representation of identification of reliable sources via database searches.

**Fig. 2.**
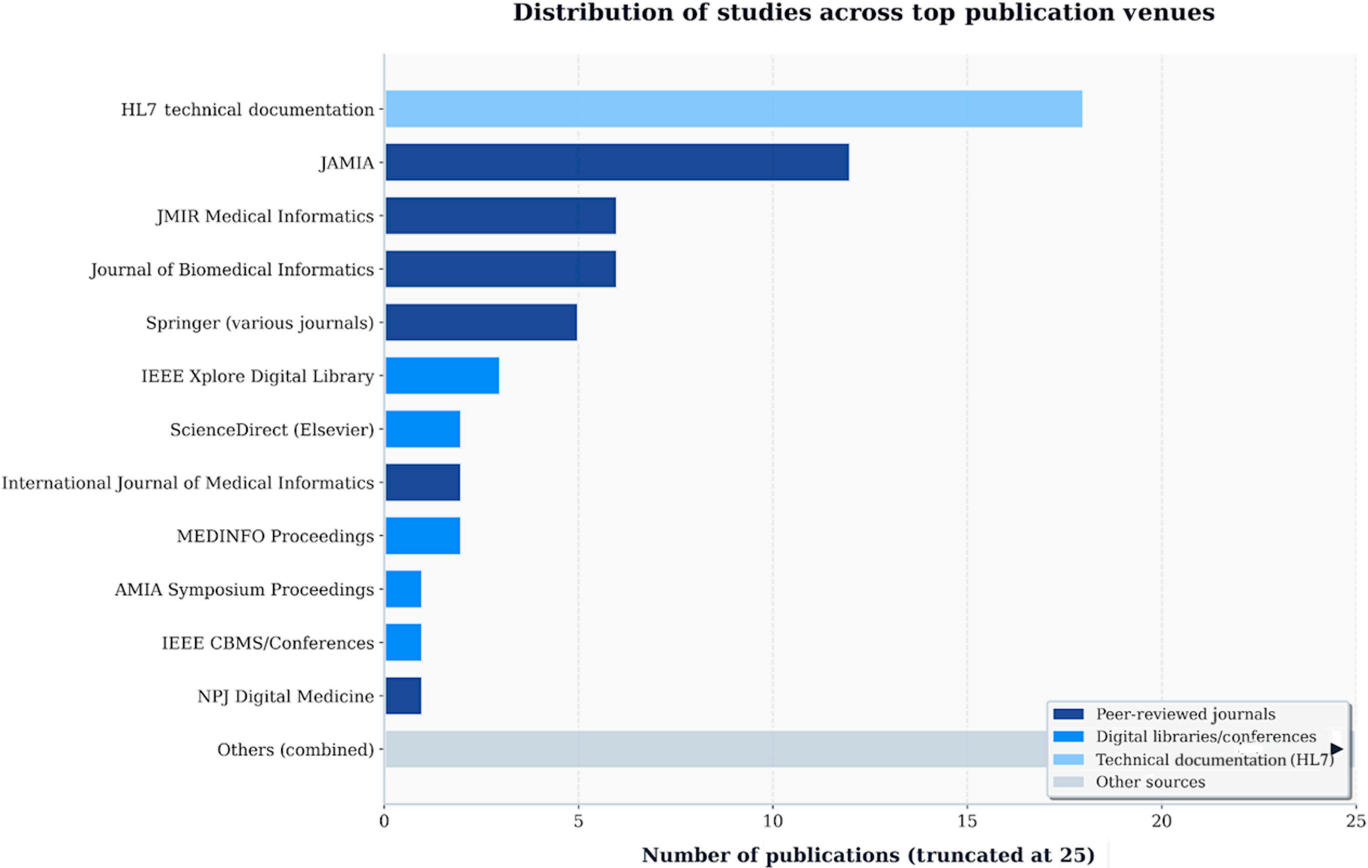
Distribution of included studies across publication venues.

A systematic search of the literature was performed in major biomedical and technical databases to identify relevant literature. The overall study selection process is illustrated in Fig. 1. The primary literature sources were PubMed/MEDLINE, IEEE Xplore, ScienceDirect, SpringerLink, JMIR, and JAMIA, which are the most important literature repositories related to clinical informatics, healthcare interoperability, health information systems, and interoperability standards. The distribution of the included studies across publication venues is shown in Fig. 2. Authoritative technical and implementation-oriented information was additionally sought from institutional and standards-based repositories such as the official HL7 International documents, technical guidance for implementation, and publicly accessible documents from national regulatory agencies.

A database-specific search strategy was created, with controlled vocabulary terms and free-text keywords related to EHRs, HL7 v2, HL7 v3/CDA, FHIR, interoperability, semantic interoperability, and healthcare data exchange. Boolean operators (AND, OR) were used to build flexible database search queries. The basic Boolean search logic, which is used throughout the review, is shown below:

(“HL7 v2” OR “HL7 version 2” OR “HL7 v3” OR “Clinical Document Architecture” OR “CDA” OR “FHIR”)

AND

(interoperability OR semantic interoperability OR health information exchange)

AND

(“electronic health records” OR “EHR” OR “health information systems”)

**Fig. 3.**
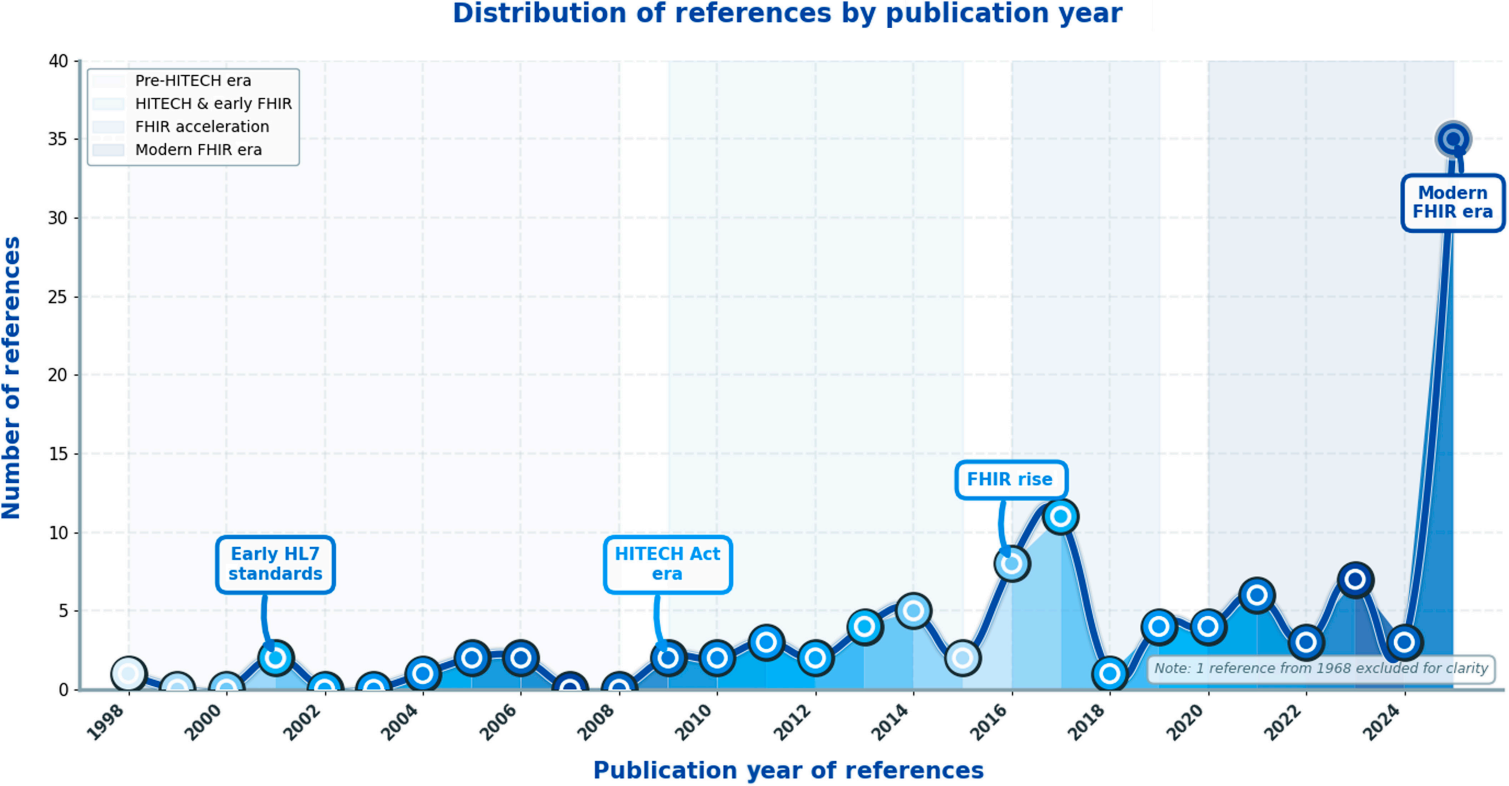
Year-wise distribution of included studies.

The full database-specific search terms for PubMed/MEDLINE, IEEE Xplore, ScienceDirect, SpringerLink, JMIR and JAMIA are listed in Table S1. The search time frame covered the early years of development of EHRs, all the way to October 2025, allowing for the inclusion of both past and current literature on EHR interoperability. The year-wise distribution of the included studies is presented in Fig. 3. The first search resulted in 422 records of peer-reviewed publications, technical documentation, and selected gray literature sources were found and cataloged for deduplication, screening, and eligibility assessment.

### Deduplication

All 422 identified records were deduplicated using a detailed deduplication process, which removed both exact duplicates in bibliographic fields and overlaps in content. This phase addressed instances in which substantially similar material appeared across multiple publication formats, including conference papers later expanded into journal articles, draft and finalized technical reports, and gray literature republished under institutional repositories. A multilevel approach that involves manual review and automated similarity matching using textual similarity and fuzzy matching methods was adopted to detect near duplicate records that exhibit strong similarity in authorship, publication timelines, or keywords. Wherever there was overlap between the flagged records, these were compared in a relatively systematic fashion and the best, and most complete version was preserved. This process assured that the final evidence base was made up of unique technical contributions, and redundancy was reduced before screening and full-text eligibility assessment.

### Screening and eligibility assessment

After deduplication, 374 unique studies were left for title and abstract screening. Screening was performed using predefined inclusion and exclusion criteria emphasizing substantive technical, architectural, semantic, or implementation-oriented discussion related to EHRs, HL7 v2, HL7 v3/CDA, or FHIR interoperability frameworks. Studies limited exclusively to clinical outcomes, usability evaluation, nontechnical health information technology (IT) discussions, or opinion-oriented commentary were excluded. Multiple authors independently reviewed screening decisions, and in the event of disagreement, decisions were made by discussing and reaching a consensus. This process eliminated 206 records, and 168 studies were available for additional full text evaluation and thematic synthesis.

### Data extraction and quality appraisal

Detailed full-text eligibility assessment was then conducted on the remaining studies, and institutional libraries, open-access repositories, and publishers’ websites were used to retrieve all manuscripts, technical appendices, implementation documentation, and supporting materials in an attempt to reduce retrieval bias. Records were assessed based on predetermined criteria that focused on technical relevance, interoperability focus, and immediate applicability to healthcare interoperability standards. The main reasons for exclusions during this stage were scope mismatch of studies, such as studies that were not directly related to interoperability architectures and standards-based implementation frameworks, and conceptual or theoretical publications that did not have meaningful technical or implementation-oriented validation. After this assessment, 107 studies made it into the final body of evidence.

Structured extraction forms were used for the extraction of relevant data from the included studies, with the aim of thematically analyzing the various and heterogeneous technical literature. Data gathered included the publication metadata, interoperability architectures, semantic representation models, implementation environments, technical specifications, conformance approaches, regulatory considerations, documented limitations, and challenges in implementing EHR systems, HL7 v2, HL7 v3/CDA, and FHIR. This was a structured extraction process to aid subsequent thematic synthesis and comparative analysis, presented below.

### Comparative thematic synthesis methodology

A comparative thematic synthesis approach was used to analyze the included studies across successive generations of healthcare interoperability standards. The synthesis focused on recurring technical themes associated with interoperability architectures, semantic representation models, implementation methodologies, conformance frameworks, scalability, terminology integration, and interoperability limitations across EHR systems, HL7 v2, HL7 v3/CDA, and FHIR. Studies were comparatively examined to identify how later interoperability frameworks addressed architectural and semantic constraints observed in preceding standards while simultaneously introducing new implementation and governance challenges. The synthesis additionally incorporated implementation-oriented evidence, regulatory guidance, and standards-based technical documentation to support a longitudinal and technically informed interpretation of interoperability evolution across heterogeneous healthcare ecosystems.

## EHRs and the Foundations of Healthcare Interoperability

The road to healthcare interoperability starts with computerization of the patient record. The EHRs have become the fundamental data source and the central subject of all data-exchange standards. Understanding its functions, and the technological and regulatory context in which it emerged, is therefore an essential starting point. However, it was not digitalization that solved the old fragmentation, despite the early EHRs being sometimes limited to a single hospital, vendor-specific or not uniform in gathering and sharing information. These constraints showed the difference between the electronic data storage and the real system-to-system coordination, which led to decades of efforts on standards and free movement of data. In that regard, a modern EHR should be perceived not only as a substitute of the paper charts but also a digital space that will accommodate a variety of clinical and administrative data, and the clarification of what an EHR consists of, does, and controls is an inevitable initial step as these traits predetermined its initial achievements as well as the interoperability issues that arose.

### Defining the modern clinical record: Core functions and data domains of EHRs

An EHR is, in general, a computer-based, longitudinal system of patient health information maintained over time by healthcare providers, which grew from problem-oriented medical records in the 1960s and hospital information systems in the 1970s [1]. The literature defines EHRs as having administrative and clinical data that are relevant to patient care, such as demographics, diagnoses, medications, progress notes, laboratory observations, and vital signs [21]. This idea is different from the earlier term electronic medical record (EMR), which was a reference to the clinical records held within a single healthcare organization [21]. Previous publications on the development of healthcare information systems have discussed the movement from EMR to EHR as a move from individual institutional record systems to more integrated and patient-centered information environments that facilitate continuity of care and interoperability across different institutions [1,21]. This interoperability goal was also strengthened by policy efforts, including the U.S. Health Information Technology for Economic and Clinical Health (HITECH) Act of 2009 that linked the “meaningful use” of certified EHR technology to the capability of interorganizational healthcare information exchange [22].

The literature reviewed also reveals some key functional areas that are expected to be supported by modern EHR systems in modern healthcare systems, such as patient records, medications, clinical documentation, medical histories, and external clinical documents [21]. As the digitization and interoperability of healthcare continue to grow, modern EHR systems are increasingly incorporating features of allergy and immunization registries, laboratory and imaging results, care coordination workflows, and clinical decision support capabilities [2]. EHR systems are now widely utilized in interwoven healthcare settings to enable information sharing among providers, laboratories, pharmacies, payers, public health systems, and patient’s digital devices, among others [23]. The literature also references that current EHR deployments also have a growing capability to support structured representation of social determinants of health to incorporate socioeconomic, behavioral, and environmental information into clinical care and population-level analyses [18]. The increasing number of healthcare data streams has greatly increased the need for semantic interoperability using standardized vocabularies like SNOMED CT, LOINC, RxNorm, ICD and more, to ensure that clinical information is interpreted correctly across all the various and diverse systems [18,23].

Research also shows that patient engagement features, such as patient portals, secure messaging, and record downloading, have revolutionized the EHR landscape from a clinician-centric system to a comprehensive healthcare communication system [24]. All of these developments are part of the evolution of the modern clinical record to be more than just a digital “paper” record, but an interactive environment of interoperability across several stakeholders, multiple sources of data, and increasing channels for communication [23,24]. At the same time, the literature surveyed indicates that this development is related to the increasing technical demands on scalability, interoperability governance, standardization, and secure data exchange, further highlighting the need for flexible, interoperable healthcare infrastructures that can manage the transmission of vast amounts of clinical data across a range of heterogeneous healthcare systems [25].

### Architectural paradigms: From on-premises monoliths to cloud-based platforms

EHR systems architecture has experienced substantial changes and transitions that have contributed to the current interoperability standards, and there are 2 dominant deployment models, local/in-house and cloud-based [21]. Digitization began with early EHRs of the 1960s to 1980s, which were built on small mainframes [3]. This was followed by the rapid introduction of on-premises commercial systems from vendors such as Epic, Meditech, and Cerner in the 1990s that solidified siloed and institution-specific data environments and delayed interoperability [1,4]. These monolithic deployments provided localized control and offline functionality at the expense of intensive IT maintenance, expensive licensing as well as affirmed weaker backup and disaster recovery [21]. Those also resulted in high vendor lock-in [26], limited system innovation and upgrades, and limited the use of EHR data for research and population health—constraints that have been reiterated in systematic reviews [4]. Comparative assessments also indicated that on-premises systems were behind the cloud architectures in governance, resilience, and recovery potentials [27].

Since 2010, the move to cloud-based EHRs has been increasing faster, and it has been triggered by the policies such as the HITECH Act in the U.S. and similar digitization initiatives in Europe and Asia [28]. Subscription-based cloud models have long-term benefits as shown in cost–benefit analysis that is based on reduced infrastructure and staffing needs [29]. It is also reported in scoping reviews that there is better collaboration, mobile access, and backup security in cloud deployment, but persistent issues continue to arise regarding latency, vendor reliance, and regulatory compliance such as the Health Insurance Portability and Accountability Act (HIPAA) and the General Data Protection Regulation (GDPR) [23,30]. The adoption is still not globally even with low- and middle-income regions preferring the hybrid or localized solutions as a result of bandwidth, financial, and policy constraints [31]. The current type of hybrid architecture is a transitional architecture that can be used between local servers or edge computing devices with workloads that are latency-sensitive, and cloud computing platforms to store and analyze data; Internet of Things wearables and bedside monitors often work in this mode, sending data to the cloud but ensuring operational continuity [32]. This type of Secure Edge of Things system continues to show how hybrid EHRs can extend further into telemedicine and home-based monitoring [32].

It is this architectural migration, from the closed on-premises system to the distributed and web-native platforms, that has directly facilitated the present-day API-based interoperability [17,31]. Cloud infrastructures are naturally compatible with RESTful models, which decrease the complexity of the legacy messaging protocols and supports the quick adoption of FHIR [17,31]. Systematic reviews confirms that FHIR RESTful architecture is closely linked with cloud-native principles, which have a better seamless interoperability, as compared to HL7 v2 or v3 [17,31]. Based on this, EHRs have moved past institutional record silos, to patient-centric, web-accessible platforms designed for exchange, analytics, and innovation-based resources, which serve as the technical basis to the subsequent policy-driven interoperability mandates [17,28,31].

### The interoperability imperative and the role of promoting interoperability programs

Mere digitization of health records was widely recognized in the literature as insufficient for improving healthcare delivery unless information could also move securely and effectively across systems and institutions. To address this challenge, policy frameworks by governments and regulatory bodies have been put in place to stimulate the uptake of interoperable health information technologies. “Promoting Interoperability Programs” (formerly the Medicaid EHR Incentive Programs) have been created in the U.S. to incentivize hospitals and clinicians to adopt, implement, upgrade, and demonstrate the “meaningful use” of certified EHR technology [21]. The HITECH Act of 2009 had a substantial impact on this policy shift and provided over 30 billion dollars to spur the adoption of EHRs and create a framework for interoperable exchange of healthcare information [3,22]. Multiple studies reported that hospital EHR adoption increased from below 10% to more than 80% within approximately 5 years [4,24], demonstrating the substantial impact of policy-driven financial incentives on healthcare digitization [4,24]. However, the literature that was reviewed all emphasized the importance of interoperability, not just digitization, as a key long-term goal, as isolated EHR deployments could generate many of the issues that were previously identified with paper-based healthcare systems [22].

The literature also depicts the “meaningful use” as a policy framework that will gradually increase interoperability capabilities across the healthcare environment. Stage 1 was largely about building basic electronic data-capture functions, such as structured documentation of medications, allergies, laboratory results, and demographics to enable patient access to their EHRs [31]. In Stage 2, structured health information exchange, enhanced clinical processes, and quality improvement using certified EHR technology systems were highlighted. There were several empirical studies that found that organizations who met Stage 2 showed improvements in patient safety indicators, and increased the amount of structured clinical data exchange, though results were mixed across healthcare settings [26,33,34]. Stage 3 subsequently shifted attention toward population health reporting, advanced clinical decision support, and patient-facing API access capabilities that later aligned closely with FHIR-based interoperability models [18,35]. The studies focused on implementation, however, always expressed concerns about administrative burden, disruption of workflow, and “checkbox compliance”, with many clinicians stating that the reporting process often compromises practical clinical usability [4,29].

The Certified Health IT Product List was created by the Office of the National Coordinator for Health Information Technology (ONC) to assess compliance with functional and interoperability requirements for certification and interoperability benchmarking [21]. However, a number of analyses found that even the certification did not ensure the real-world interoperability [30,32], because the interpretation of the implementation requirements was different among vendors and many systems still exhibited inconsistent real-world interoperability results [30,32]. The literature that was reviewed also linked HITECH and Meaningful Use incentives to quick vendor-market consolidation and the creation of what some researchers refer to as an “EHR monoculture”, that was viewed by supporters as promoting standardization, but by critics as vendor lock-in and dampening innovation [26,36]. Additional studies also indicated that smaller clinical practices, safety-net facilities, and rural hospitals were likely to encounter more challenges in interoperability participation, highlighting potential digital divides between access to interoperability within health systems [3,37].

There are also some unintended consequences noted in the literature with regard to Meaningful Use and Promoting Interoperability initiatives. Although metrics of interoperability improved measurably, several studies reported a substantial rise in documentation burden, disruption in workflow, and clinician burnout after widespread adoption of EHRs [4,29]. Other studies noted that technology for exchanging information existed in many healthcare organizations, but due to various competitive and organizational concerns, they chose not to share data, as referenced in the literature as “asymmetric interoperability” [35]. Comparative studies of public and enterprise health information exchanges also found the priority of its organizations to be different, with community-focused exchanges more interested in a more general approach to sharing information and enterprise exchanges often strengthening institutional barriers [38]. At the same time, EHR-generated metadata and audit logs created new opportunities for studying clinical workflow patterns, teamwork, and cognitive workload within digital healthcare environments [39]. Overall, the evidence reviewed indicates that these policy efforts have been important drivers of standards-based interoperability and helped to shape the emergence of API-driven exchange models like HL7 FHIR, notably via Stage 3 interoperability requirements [18,22,38]. However, the issues with data silos, vendor lock-in, operational costs, administrative burden, and data privacy remain as ongoing obstacles to more open, scalable, and interoperable healthcare data exchange models [29,37].

### Persistent challenges and limitations in EHRs

Despite the fact that the implementation of EHRs has enhanced clinical decision support, increased patient engagement, and improved access to health information, implementation has continued to reveal intractable gaps. Persistent fragmentation of healthcare data environments, in which patient data remain confined to isolated institutional systems or proprietary vendor platforms, continues to prevent the formation of longitudinal health records and coordination across care settings [7,40,41].

The second major weakness is usability. Decades of evidence demonstrates that interfaces that are not well designed, and documentation that is rigid, use too much clinician time, increase cognitive load, impair workflow efficiency, and negatively impact the quality of interactions between patients and clinicians. Such burdens, in turn, contribute to professional dissatisfaction and have been repeatedly linked to higher burnout levels [8,10,42,43]. To add to this, there are structural and economic obstacles, the high costs of implementation and maintenance are skewed against smaller organizations, and the need to rely on vendors to create an ecosystem increases costs, removes flexibility, and entrenches market concentration, ultimately sweeping innovation and interoperability across systems into slower rivers of change and progress [7,44,45].

Another instability issue is the security and privacy concerns. EHR systems have become appealing targets for cybercrime due to the digitization of sensitive health information, and various studies have reported the vulnerabilities of access control, data protection, and system governance; massive breaches testify to the fact of their importance [12,46,47]. Patient fears on confidentiality still exist especially where data sharing is not limited to direct clinical treatment [48]. Combined, these unaddressed limitations indicate that digitization is not enough. Integrity of siloed systems, usability issues, financial limitations, and vulnerability of privacy necessitate standards-based interoperable infrastructure that will precondition the creation of HL7 and FHIR as the basic frameworks of secure and scalable health data exchange [12,46].

**Fig. 4.**
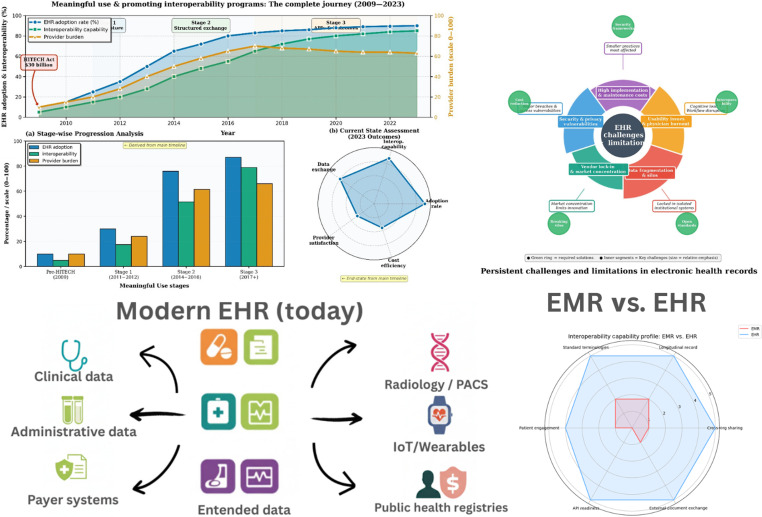
Synthesized overview of electronic health record (EHR) evolution, interoperability initiatives, implementation challenges, ecosystem integration components, and comparative interoperability characteristics associated with electronic medical record and EHR environments. The comparative visual representations included in this figure reflect qualitative synthesis-based interpretation derived from the reviewed literature rather than quantitative benchmarking measurements. Source: Authors’ synthesis based on reviewed interoperability literature and technical documentation.

Figure 4 summarizes the major interoperability evolution trends, implementation characteristics, ecosystem integration components, and comparative EMR–EHR distinctions discussed throughout the reviewed literature.

## The Genesis of a Standard: The History and Architecture of HL7 v2

Prior to the development of the standardized interoperability frameworks, healthcare data exchange was largely dependent on costly institution-specific interfaces that involved custom integration between healthcare organizations and vendors, reducing wider health IT interoperability. In the reviewed literature, HL7 v2 is a prominent answer to this fragmentation and has reached the most widespread level of adoption in the healthcare sector, largely due to its relatively lightweight yet extensible design utilizing delimiters, segments, and event-driven healthcare workflows. Research often links the broad uptake of HL7 v2 with its modular design, backward compatibility, and flexibility of site-specific customizations, allowing healthcare organizations to modernize their systems without having to sacrifice investment in the infrastructure they already have. This implementation flexibility contributed substantially to its long-term operational relevance, particularly in healthcare environments where continuity and system stability were prioritized.

Concurrently, literature reviewed repeatedly indicates that there was a great deal of semantic variability and inconsistencies in interoperability between systems as a result of extensive local customizations, inconsistent use of optional fields, and vendor-specific implementation practices. Consequently, HL7 v2 is widely portrayed not only as a transformative interoperability framework that accelerated large-scale healthcare information exchange but also as a source of many of the standardization and semantic consistency challenges that later interoperability models attempted to address.

### From ad hoc interfaces to a consensus standard: The origins of Health Level Seven

The origins of HL7 trace back to the fragmented hospital systems of the late 1970s to 1980s, where mainframe financial and registration platforms operated separately from emerging departmental systems, creating “islands of automation” and an urgent need for direct data exchange [7]. The immediate precursor was the UCSF Medical Center protocol (1981), which pioneered an application-level (OSI Layer 7) interchange model; the name “Health Level 7” directly reflects this focus on data semantics rather than transport [7]. Its modularity aligned with parallel efforts such as ASTM E1238 and ANSI X12, making it compatible with broader administrative and laboratory standardization movements [11].

At this time, researchers like Dr. Clement McDonald defended Clinical Data Interchange standards, comparing them with the transformational Universal Product Code system, but they were initially skeptical of such proposals by clinicians who were resistant to healthcare computerization [7]. Early U.S. federal funding programs supported the significance of structured data exchange and placed standards as a base of infrastructural health information policy in the nation [5]. All these strands came together in 1987 as the HL7 organization was formed out of an ad hoc group to a full standards development organization [7,49]. HL7 v1 and v2 improved the UCSF protocol and while adopting ANSI X12 and ASTM E1238 ideas and concepts and the pragmatic, implementation-driven evolution allowed it to be adopted nationally and globally in a short period of time [7,49].

Now that the historical context has been set, the following subsection will look at the construction of HL7 v2 messaging: its parts, boundaries, and triggers and how these elements will aid in scalable communication that is dependable in the heterogeneous healthcare settings [7].

### The HL7 v2 messaging paradigm: A technical deep dive

All of the literature reviewed describes HL7 v2 as a “low complexity”, practical, and “event-driven” interoperability standard that was able to facilitate large-scale communication across heterogeneous healthcare systems [8]. HL7 v2 was conceived as a system for exchanging operational information as clinical activities happened, rather than as a static transfer of data records [8]. Clinical trigger events for the operation of the system were envisioned to include patient admission, reporting of laboratory results, and order processing [8]. This workflow-based design is often linked to the use of HL7 v2 by most hospitals, laboratories, pharmacies, and administrative systems due to its strong interworking capabilities with the legacy infrastructure and the existing workflow in the institutions [8].

The reviewed studies also show that the hierarchical and delimiter-based organization of HL7 v2 messages was helpful to its scalability, but also caused a number of its interoperability issues over time [7,8]. This flexible arrangement of segments, fields, components and subcomponents allowed institutions to share a variety of segments and information types from both the clinical and administrative domains and to tailor implementations to their local operational needs [7,8]. The same implementation flexibility also resulted in substantial differences in implementation across healthcare settings. Semantic inconsistencies, parser incompatibilities, and higher interface maintenance needs across vendors and healthcare organizations were often reported in multiple investigations, due to the use of optional fields, institution-specific customizations, and inconsistencies in the interpretation of message structures [7,8].

The results of the provided studies also demonstrate the use of the Minimal Lower Layer Protocol (MLLP) in the transmission of HL7 v2 messages over Transmission Control Protocol/Internet Protocol-based clinical networks [44]. Multiple implementation-related analyses noted that acknowledgment in MLLP increased the likelihood of successful transmission and decreased the likelihood of message duplication in clinical workflows with high message volume especially in admission–discharge–transfer and laboratory information systems [46]. Meanwhile, the literature reviewed often highlighted that the tightly coupled encoded nature of HL7 v2 messages often necessitated the use of specific interface engines and middleware platforms to transform, route, validate, and handle errors of messages between disparate systems [8,50].

It has also been reported that the adoption of character encoding standards like UTF-8 played a substantial part in the global deployment of healthcare systems, as it allowed for the exchange of patient information in multiple languages, in geographically distributed healthcare systems [51]. However, many reviewed studies argue that the extensibility and customization capabilities of HL7 v2 simultaneously reinforced localized implementation practices that complicated standardized interoperability and increased long-term integration overhead [7,50]. Overall, the literature included suggests that HL7 v2 is a very influential interoperability framework, where implementation flexibility was a key factor in its massive adoption, but this flexibility has also led to semantic inconsistencies and to fragmentation of interoperability in the healthcare ecosystem [7,8,50].

### Anatomy of a message: Detailed analysis of the MSH and PID segments

Based on the reviewed literature, the Message Header (MSH) and Patient Identification (PID) segment are identified as essential operational components of HL7 v2 messaging: They play a critical part in the routing, interoperability coordination and patient identity management of the distributed healthcare environment [8,46]. Studies examining institutional HL7 deployments frequently report that the MSH segment enabled reliable message interpretation, acknowledgment handling, and transaction management across heterogeneous clinical systems through standardized metadata structures associated with message routing and version identification [8,43]. The reviewed studies also suggest that these capabilities played a vital role in the early of scalability of HL7 v2 in growing hospital, laboratory, pharmacy, and administrative information networks, especially in scenarios where the interoperability of systems developed independently and legacy infrastructures required to be accomplished [43].

The literature also describes the PID segment as being extremely important for disseminating demographic and patient identity information among healthcare organizations [46]. According to multiple investigations, patient identification workflows based on HL7 v2 contributed substantially to the implementation of patient tracking across various systems, continuity of care, and exchange of clinical information across systems in distributed healthcare infrastructures [46]. Meanwhile, several studies with an implementation focus also noted issues with the variability of how identifier management is performed, as well as how often identifiers were assigned by different authorities and how different institutions customized identifiers with different values, all of which often complicated patient matching processes and led to fragmented patient records in systems [46]. The results are often linked to other semantic interoperability problems that arose in the implementation context in different and heterogeneous healthcare settings. The representative HL7 v2.5 MSH and HL7 v2.4 PID fields frequently referenced in interoperability and conformance studies are summarized in Table 1.

**Table 1.** Representative HL7 v2.5 MSH and HL7 v2.4 PID fields frequently referenced in interoperability and conformance studies

Field	Field Name	Type	Len	Opt	Rep	Interoperability relevance
(A) HL7 v2.5 MSH segment fields						
MSH.1	Field Separator	ST	1	R	N	Defines delimiter syntax for parsing.
MSH.2	Encoding Characters	ST	4	R	N	Specifies encoding delimiters.
MSH.3	Sending Application	HD	227	O	N	Identifies originating application.
MSH.4	Sending Facility	HD	227	O	N	Identifies originating facility.
MSH.5	Receiving Application	HD	227	O	N	Identifies destination application.
MSH.6	Receiving Facility	HD	227	O	N	Identifies destination facility.
MSH.7	Date/Time of Message	TS	26	R	N	Supports sequencing and auditability.
MSH.8	Security	ST	40	O	N	Carries security-related information.
MSH.9	Message Type	MSG	15	R	N	Defines workflow and trigger event.
MSH.10	Message Control ID	ST	20	R	N	Enables acknowledgment tracking.
MSH.11	Processing ID	PT	3	R	N	Indicates operational environment.
MSH.12	Version ID	VID	60	R	N	Specifies HL7 version used.
MSH.15	Accept Ack Type	ID	2	O	N	Defines acknowledgment behavior.
MSH.16	App Ack Type	ID	2	O	N	Specifies application acknowledgments.
MSH.18	Character Set	ID	16	O	Y	Supports multilingual encoding.
MSH.21	Message Profile ID	EI	427	O	Y	References conformance profiles.
(B) HL7 v2.4 PID segment fields						
PID.1	Set ID	SI	4	O	N	Sequence identifier within groups.
PID.3	Patient Identifier List	CX	250	R	Y	Supports cross-system patient matching.
PID.5	Patient Name	XPN	250	R	Y	Stores structured patient identity.
PID.7	Date of Birth	TS	26	O	N	Supports demographic matching.
PID.8	Sex	IS	1	O	N	Demographic classification reference.
PID.10	Race	CE	250	O	Y	Supports population-health reporting.
PID.11	Address	XAD	250	O	Y	Stores residence information.
PID.13	Phone – Home	XTN	250	O	Y	Provides patient contact information.
PID.18	Account Number	CX	250	O	N	Links records with billing systems.
PID.19	SSN	ST	16	B	N	National identity reference if permitted.
PID.29	Death Date/Time	TS	26	O	N	Records mortality timing.
PID.30	Death Indicator	ID	1	O	N	Indicates deceased patient status.

### Enforcing specificity: The HL7 v2 conformance methodology and message profiling

The studies consistently identifies the flexibility and optionality of HL7 v2 as both the primary reason for its widespread adoption and one of its most substantial interoperability limitations [52]. Although HL7 v2 enabled rapid integration across heterogeneous healthcare systems, studies repeatedly report that organizations implementing the same version of the standard frequently remained unable to exchange information consistently because of differences in optional segment usage, local field constraints, and vendor-specific coding practices [52]. This widespread implementation variability created what many researchers describe as a “non-standard standard” environment in which nominal HL7 v2 compliance did not necessarily guarantee semantic interoperability across systems [52].

To address these inconsistencies, HL7 introduced a formal conformance methodology centered on the concept of profiling, in which the base HL7 v2 specification functioned as a flexible framework rather than a rigid implementation standard [52]. The reviewed studies describe message profiles as constrained interoperability specifications tailored to particular workflows and use cases through restrictions applied to optionality, cardinality, vocabulary bindings, fixed values, and field-length requirements [52]. These constraints were typically organized within broader Implementation Guides (IGs), which provided contextual definitions, interaction sequences, workflow specifications, and interoperability requirements for specific deployment environments [52]. While profiling substantially improved implementation consistency and interoperability reliability, several studies also observed that widespread creation of local or semicustom profiles contributed to continuing fragmentation across healthcare ecosystems [52].

There has also been a growing formalization and computability of HL7 v2 conformance methodologies as shown in recent literature [53,54]. Studies of current use of HL7 reveal that the distinction between normative and informative guidance is now clearly made and that systems can now make machine-verifiable claims of conformance using profile identifiers and structured validation techniques like the MSH-21 reference [53]. Other investigations describe the emergence of tooling ecosystems including IGAMT, automated profile-authoring platforms, executable validation suites, and test-generation frameworks that transformed IGs from static documentation into computable interoperability artifacts [54]. National and domain-specific interoperability initiatives, including immunization and vital-records exchange programs, increasingly adopted hierarchical profiling approaches in which local IGs extended broader national interoperability constraints according to jurisdictional or workflow-specific requirements [55].

Conformance testing is another key element of modern-day HL7 v2 interoperability implementations, identified in the literature as well. Studies evaluating interoperability validation workflows report that organizations such as National Institute of Standards and Technology developed automated testing infrastructures capable of generating validation datasets, executable test plans, and machine-verifiable standardized validation message sets directly from computable profiles, thereby reducing manual testing effort and improving interoperability consistency at large-scale implementation events and connectathons [56]. However, some studies point out the fact that the legacy of vendor extensions, the uncontrolled proliferation of profiles, and the lack of governance creates challenges to validate interoperability and for long-term maintenance [57]. To address these risks, it is more and more suggested to implement better governance procedures such as reusable profile registries, curated terminology bindings, stability and conformance of profile identifiers, and tighter conformance requirements [57].

Other research shows that the IHE Technical Framework serves as a complementary mechanism to increase interoperability of the HL7 v2 standard by harmonizing language and using standardized profiling conventions and segment constraints for various healthcare domains [15]. The frameworks for interoperability have grown in importance in hybrid modernization approaches where well-defined HL7 v2 profiles are mapped to FHIR resources and in that way facilitate semantic consistency and minimize information loss during the move to the newer approach to interoperability [15]. In total, the evidence reviewed indicated that HL7 v2 conformance methodologies and the supporting tooling ecosystems evolved the standard from being a loose interpretation of the message exchange model into a more rigorous and computable interoperability engineering model, without losing the flexibility needed in a heterogeneous healthcare environment [53,54,56].

**Fig. 5.**
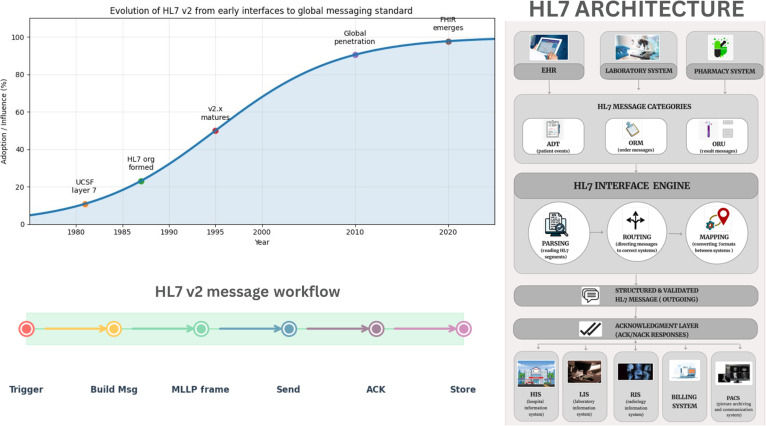
Conceptual overview of HL7 v2 evolution, trigger-event messaging workflows, interface-engine architecture, and interoperability processes within healthcare information systems. The visual representations included in this figure reflect qualitative synthesis-based interpretation derived from reviewed interoperability literature and technical documentation. Source: Authors’ synthesis based on reviewed interoperability literature and technical documentation.

As illustrated in Fig. 5, HL7 v2 interoperability environments commonly relied on trigger-event messaging workflows, interface-engine architectures, and institution-specific implementation practices, which collectively contributed to both the widespread adoption and persistent semantic variability associated with HL7 v2 ecosystems.

### The evolution of HL7 v2, its global adoption trajectory, and the emerging limitations driving the need for successor standards

HL7 v2 grew at a very fast rate during the 1990s and the early 2000s to become the most popular global healthcare messaging standard because of its event-driven architecture, its lightweight implementation, and its capability to support heterogeneous clinical processes across hospitals, laboratories, and pharmacies [58]. Its flexibility led to its extensive use in North America, Europe, and Asia-Pacific to share demographics, orders, and laboratory results [58].

However, with this elasticity there came difference. The local systems varied in the application of optional fields, segments, and sets of value, and this created interface heterogeneity necessitating a lot of customization to obtain interoperability [58]. Comparative analysis revealed that HL7 v2 was still firmly established but HL7 v3 did not enjoy a wide adoption due to its extreme inflexible modeling and steep learning curve [59].

Taken together, these trends indicate that the development of HL7 signifies the weaknesses and strengths of the previous versions. The international success of v2, the poor uptake of v3, and the rapid increase of FHIR reflect a series of improvements in which the new generation attempts to fix the weaknesses of the former one [12,58,59].

Although HL7 v2 represented a step forward in interoperability by enabling the exchange of structured messages, the shortcomings of this purely syntactic approach became evident shortly thereafter. Stakeholders realized that sustainable interoperability needed to go beyond structured transmission and entail semantic consistency among systems. This shift in thinking drove the development of semantically rich standards, resulting in HL7 v3 and the CDA, which attempted to formalize meaning and ensure that transmitted information preserved clinical intent across applications.

## A Paradigm Shift toward Semantics: HL7 v3 and the CDA

This continued interoperability inconsistency and variability of HL7 v2 led to a substantial change in a healthcare interoperability design philosophy. To address these, HL7 developed a new version, called Version 3 (v3), which had a more formal model-driven paradigm aimed at providing better semantic interoperability and meaning of clinical data across the diverse healthcare landscape [47]. Of the many parts of the HL7 v3 ecosystem, the CDA proved to be one of the most successful for structured clinical document exchange, albeit limited adoption of full HL7 v3 messaging due to the architectural complexity and implementation overhead. The literature reviewed often suggests this adoption is due to the reference information model (RIM), which offered a semantically sound way of modeling the complex clinical relationships and standardized information structures in healthcare [41]. The CDA templating mechanisms have been reported as helpful in healthcare organizations to customize documents to meet their local clinical and regulatory requirements while preserving the semantics of the much larger HL7 v3 semantic context for the CDA documents [10]. Because of this, although there were wider uptake problems that would be encountered in the clinical documentation and health information exchange environments with HL7 v3, CDA had achieved a high level of uptake. The evolution of the RIM is most apparent in the semantic transition, and was the structural underpinning for HL7 v3.

### The RIM: An object-oriented framework for healthcare

The reviewed literature identifies the RIM as the conceptual foundation of the HL7 v3 initiative and one of the earliest large-scale efforts to establish formally defined semantic interoperability within healthcare information systems [10]. In contrast to the comparatively flexible structure of HL7 v2, HL7 v3 adopted a model-driven architecture in which messages and clinical documents were derived from a unified information model intended to provide explicit semantic relationships between clinical and administrative data elements [60]. Studies examining the evolution of healthcare interoperability standards consistently describe this transition as an attempt to reduce the semantic ambiguity, implementation inconsistency, and interoperability variability frequently associated with HL7 v2 environments [47,60]. The relationship between HL7 v3 and CDA was fundamentally based on the HL7 v3 model-driven architecture, in which CDA document structures were progressively derived through the RIM → D-MIM → R-MIM → HMD → Extensible Markup Language (XML) Schema transformation workflow [10,60]. This hierarchical derivation process enabled semantically structured CDAs while preserving alignment with the broader HL7 v3 interoperability framework [10,60].

The RIM is also reported to have brought a formally structured and object-oriented approach to semantic modeling that allows to model complex clinical workflows in a consistent way, in addition to the contextual healthcare relationships modeled by the RIM [60]. This semantic formalism was considered to be one of the most important conceptual steps forward in interoperability research since it allowed to represent relations between data and processes in healthcare systems in the domain of heterogeneous systems in a more standardized way [60]. Detailed analyses of implementation, however, also found numerous times that the formalism and abstraction of the RIM added too much complexity for the developers and healthcare organizations used to the more operationally flexible HL7 v2 [47]. As a result, some of the studies describe HL7 v3 as being “semantically strict but operationally challenging” especially within healthcare systems that rely on a wide range of legacy systems and workflows that are unique to their institutions [47].

Following this study, various methods of implementing RIM-based interoperability architectures were explored and interoperability integrated with the current clinical systems [9,61,62]. The RIM was also seen as a canonical semantic framework in model-driven interoperability studies but also as a mapping effort of mapping local schemas, terminologies, and institutional data models with the constructs of the RIM [9]. Other research work studied relational mapping and ontology-based interoperability approach that would enable distributed healthcare environments to have maintainable architectures, semantic query solutions, and heterogeneous data integration [61,62]. These were also tested in clinical decision-support situations and ontology-based semantic reasoning showed superior integration and interpretation of distributed clinical data sets [63].

Nevertheless, comparison studies always show that the use of the RIM generated the challenges of higher mapping overhead, complex implementation, and lower adoption of a complete HL7 v3 messaging infrastructure [64]. The reviewed literature also mentions tooling ecosystems like caAdapter, RIMBAA workstreams, and Open Health Workbench, which were created to decrease the amount of manual mapping and to allow other systems that could not be replaced entirely, to be used interchangeably [61,62]. Despite all this, many of the studies have led to a belief that the problems of implementing the full RIM-based architectures have led to an increased interest in more deployment-oriented approaches to interoperability that are capable of providing for semantic consistency with decreased implementation problems [64]. This move later shaped the evolution of the HL7 CDA, which used RIM principles of semantics as part of structured clinical documents for reporting, regulatory documentation, and clinical information exchange across multiple systems [10,60].

### From messages to documents: The structure and principles of the HL7 CDA

While the adoption of HL7 v3 messaging was relatively small, the literature reviewed throughout the document shows that the CDA was one of the most successful and adopted aspects of the HL7 v3 ecosystem [65]. This adoption has been explained by the capacity of CDA to integrate the formal semantic structure and real-life clinical documentation needs, especially in clinical setting where the need is for a persistent, human-readable, and legally valid clinical record [65,66]. Unlike transaction-oriented interoperability methods, CDA was created with the notion of the clinical document being complete and contextually persistent information object, one that can be stored for a long time, be subject to compliance, and can be used for clinical communication across institutions [65,66].

The literature reviewed consistently points to the following properties that facilitated the implementation of CDA: persistence of documents, stewardship, legal authentication, document wholeness, and human readability [66]. Of these, it is generally recognized that the human readability aspect is a substantial practical benefit as it allowed clinicians to interpret the information that was exchanged even when the systems they received the information from were not able to process all of the structured semantic elements [66]. According to several research works, this capability is described as a highly important “transitional mechanism” enabling healthcare organizations to join in a structured way in document exchange without the need to fully implement all of the highly complex semantic infrastructures [66]. Consequently, CDA became widely adopted in discharge summaries, continuity-of-care documents, referral communication, and regulatory reporting workflows where both machine-processable structure and clinician-readable presentation were required [65].

Research analyzing the deployment of CDA also indicates that the architecture of CDA was used to integrate structured coded entries with narrative clinical documentation based on local workflow needs and interoperability needs of the healthcare organizations [65,66]. This flexibility is often cited in the literature as a key element in supporting adoption in heterogeneous healthcare settings, as it enabled institutions to retain clinically relevant narrative information while providing a means for enhancing the ability to structure the information semantically and to share it effectively with others [65]. Concurrently, implementation analyses reported some interoperability inconsistencies between institutional deployments due to the lack of quality in documentation practices, and the variance in semantic structuring [65,67].

All the studies included share a common theme on the part of the CDA templating and validation mechanisms in enhancing semantic consistency and interoperability reliability between different implementations [67,68]. The literature cites CDA templates as useful constraint mechanisms that can be specified using structural rules, terminology bindings, and expectations of validation to achieve consistency of implementation across CDA models [67]. Consolidated CDA has been described as a big standardization effort, as it offered a set of reusable vendor-neutral templates and also automated validation methods designed to minimize implementation variability [68]. Further, research assessed the CDA information exchange validation workflows, which showed that using structural validation, terminology validation, and semantic quality assurance in layered validation processes generated more correct documents and achieved higher semantic fidelity in information exchange [69]. Meanwhile, tooling environments like Model Driven Health Tools were created to ease the implementation effort by automatically generating schemas, providing schema validation support, and managing the schemas using templates [70].

Literature reviewed also showed that CDA was not only relevant in a direct exchange situation of questions and answers but was also relevant beyond that. The transformation of CDA documents to research-oriented data models, like the Observational Medical Outcomes Partnership Common Data Model, has been investigated in a number of ways, including the use of terminology mapping, template-aware parsing, and narrative postprocessing methods [71]. The other interoperability studies focused on hybrid CDA–FHIR architectures, where legacy CDA documents were interfaced with modern FHIR resources like *DocumentReference* and *Composition* to enable the step-by-step transition toward API-based interoperability, while maintaining the authenticity of the documents and legal traceability [72]. Meanwhile, the practical implementation experience also highlighted issues with template governance, lack of consistent terminology, a disjointed tooling landscape, and high remediation costs [68,69], thus pushing for increased adoption of validated consolidated CDA models and interoperability governance models [68,69].

Overall, comparative evaluations of the studies analyzed tend to show that CDA is generally very effective in documentation tasks involving clinicians, in preserving longitudinal records, in legal archiving, and in structured clinical reporting, while highlighting the barriers to the support of fine-grained and low latency interoperability with high transactionality requirements of contemporary API-based healthcare systems [72,73]. Consequently, the literature now speaks of the complementary nature of the 2 interoperability standards, CDA supporting authenticated exchange of documents and FHIR allowing for access to the resources in a granular, modular, and real-time fashion in today’s digital health ecosystem [72,73].

### Technical implementation: The role of XML and templated constraints

XML-based encoding is a key feature of CDA found in the reviewed literature, as it allowed formally structured and machine-processable clinical documents, based on the HL7 v3 RIM semantics and HL7 v3 data types [65]. The findings from the studies on the implementation of CDA consistently show that it led to greater consistency of documents and enhanced the semantic interoperability between heterogeneous healthcare systems, especially in such systems where the longitudinal clinical information needs to be exchanged in a standardized manner across the various systems [65]. Meanwhile, a few studies found that the semantic formalism and validation of the validation requirements in XML-based CDA implementations made it more difficult to implement and integrate with less complex messaging-oriented interoperability methods [65].

All of the existing studies demonstrates the importance of CDA templating mechanisms as a way of making the general CDA framework suitable for different interoperability needs on the clinical and organizational levels. Like the profiling approach in HL7 v2, a CDA templating approach was tasked with providing structuring and semantic constraints that could be reused across different CDA implementations to ensure consistency and reliability of interoperability across implementations [65]. Researchers have consistently found that one of the most important reasons for maintaining semantic consistency across diverse document types in real-world deployments of CDA is the use of a template for the documents, such as the U.S.-specific Continuity of Care Document IG [65]. The studies further notes that template identifiers and modular template libraries supported localized customization while preserving alignment with broader CDA semantic frameworks and interoperability objectives [65].

Within the literature, there is a distinction between 2 types of CDA implementations: open and closed templating approaches [65]. Comparative implementation studies show that open and closed templating approaches were successful in expanding the document structures to meet local operational needs and in providing semantic consistency and conformance to the structure, respectively [65]. This adaptability was seen to lead to greater uptake in heterogenous healthcare settings, but some studies also reported that interoperability issues arose due to variability in the way the template was implemented in systems [65]. As a result, literature reviewed here has focused on the need for formal template management, conformance methods, and validation procedures that will enhance the semantic reliability of the exchanged documents and minimize the variance in implementation between different document exchange scenarios [74].

Another major focus of interest among the studies included are validation and semantic enforcement mechanisms. In addition to usual XML schema validation, researchers were more and more turning to the use of the higher-order schema validation frameworks for the semantic level checking and the use of the template-driven schema validation framework Schematron for the structure and the terminology bindings conformity assessments [75]. The studies on semantic interoperability also highlighted the need to have a standard vocabulary like SNOMED CT, to ensure semantic consistency and minimize ambiguity when exchanging clinical documents across systems [76]. Other investigations delved into ontology-based integrity checking methodologies designed to enhance the consistency of the use of coded clinical concepts and to enable more precise semantics interpretation in various and heterogeneous healthcare systems [76].

Security and document authenticity were also mentioned as key factors to consider in CDA implementations. A number of studies were conducted on XML Digital Signature mechanisms that allowed for selective redaction of sensitive information, while ensuring the legal validity and integrity of clinical documents authenticated by the digital signature [77]. These capabilities are often linked to an environment where compliance, auditability, and controlled disclosure of protected health information is required, such as in the healthcare sector [77].

The overall literature suggests that CDA templating and validation methods are necessary to ensure the consistent meaning of the CDA in a variety of diverse healthcare settings while allowing for flexibility during implementation. Although CDA’s XML-based structure and semantic constraints enabled highly structured and legally reliable clinical document exchange, the reviewed studies repeatedly emphasize that effective interoperability depended not only on document encoding and validation but also on consistent interpretation of clinical terminology across systems [74–76]. The increasing need for common terms and for standard semantic interpretation further insured the role of terminology services, interoperability standards based on ontologies, and semantically controlled healthcare data exchange architectures in subsequent interoperability standards.

### Semantic interoperability in HL7 v3: Role of vocabularies, terminologies, and code systems

The literature reviewed suggests that HL7 v3 is a key milestone in the evolution of syntactic interoperability to semantically accurate healthcare information exchange, in the context of controlled vocabularies and ontology-based terminology systems. The use of standards like SNOMED CT, LOINC, and ICD were meant to facilitate the understanding of the clinical concepts within highly diverse healthcare settings and to minimize semantic inconsistencies found within locally established code systems in HL7 v2 implementations [1,10,66] This semantic shift went beyond simple data transfer to semantically more similar interpretation of the clinical information within different systems and institutions [1,10,66].

They also demonstrate that SNOMED CT allowed for a very granular clinical coding, LOINC for a uniform coding of laboratory results and observations, and ICD for a uniform reporting of morbidity and mortality events across country and internationally [4,18,26,31]. These vocabularies became more and more used to act as a semantic glue that enhanced consistency and minimize the fragmentation of the interoperability across the health system [2,22,24]. Despite this, there are also many difficulties in terms of terminology governance, ontology alignment, mapping complexity, and harmonization of the various coding systems in use [27,38]. Semantic interoperability in a HL7 v3 environment was still not fully realised because of the differences in institutional and regional adoption of this standard [22,39].

Recent research further indicates that maintenance of terms, ontologies, and scalable machine-readable terminology services are essential for long-term success of semantic interoperability [4,18,78]. Together, these advances made interoperability a computable semantic framework that could enable building clinical reasoning, analytics, and learning health systems [1,26,31]. Meanwhile, the complexity of these semantic models, both in terms of their operational and structural complexity, led to the development of CDA and FHIR that sought to maintain the concepts and semantic consistency while offering greater flexibility and scalability in implementation.

**Fig. 6.**
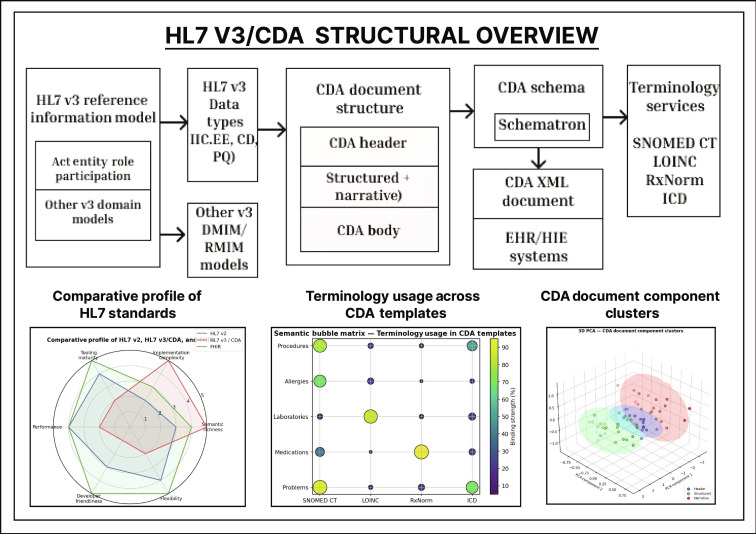
Synthesized structural overview of HL7 v3/Clinical Document Architecture (CDA) interoperability architecture, semantic modeling, terminology integration, and validation frameworks. The comparative visual representations included in this figure reflect qualitative synthesis-based interpretation derived from the reviewed literature rather than quantitative benchmarking measurements. Source: Authors’ synthesis based on reviewed interoperability literature and technical documentation.

As illustrated in Fig. 6, HL7 v3 and CDA introduced semantically structured interoperability models centered on the RIM, standardized document architectures, terminology integration, and schema-based validation mechanisms intended to improve semantic consistency across healthcare systems.

### HL7 v3/CDA in practice: Adoption barriers, critiques, and comparisons

While the HL7 v3 and the CDA have made substantial strides toward semantic interoperability, the literature reviewed indicates that these frameworks have substantial barriers to implementation and adoption. Though conceptually solid, the RIM was architecturally complex and demanded specialized expertise and was hard to deploy in many healthcare settings, leading to uneven and regionally variable adoption patterns [79]. Studies also show that even when the CDA system is certified, the system often did not conform to the CDA standards, it did not always use the same terminology to identify the same concepts, and validation errors often occurred repeatedly, needing manual reconciliation, falling short of the original vision of the CDA system for semantically interoperable systems [80].

Other factors, such as performance and tooling, limited wider adoption. Some studies reported that the detailed XML structure and the multilayer validation processes involved with CDA—schema validation, Schematron rules, and terminology verification—added substantial computational burden, making it less suitable for real-time transactional interoperability while remaining well suited for document-oriented workflows such as discharge summaries and referral letters [81]. Although tooling ecosystems such as Model Driven Health Tools and caAdapter reduced portions of the implementation burden, they never achieved the ecosystem maturity, usability, or developer adoption observed in HL7 v2 or later FHIR-based infrastructures, thereby contributing to persistent implementation complexity and higher operational costs [82]. At the same time, the widespread institutional dependence on HL7 v2 limited large-scale migration toward fully CDA-centric architectures, leading many organizations to adopt hybrid interoperability environments in which HL7 v2 continued supporting transactional exchange while CDA was primarily used for structured document sharing [83]. Together, these restrictions led to the development of HL7 FHIR, which overcomes many of the complexity, performance, and tooling issues of HL7 v3/CDA implementations through its resource-oriented design, RESTful APIs, and lightweight serialization capabilities like JavaScript Object Notation (JSON) [84]. In general, the reviewed literature paints a picture of transition to FHIR as an evolution from highly formal, but more operationally complex interoperability frameworks to more flexible, scalable, and developer-friendly interoperability ecosystems that are more closely aligned with current healthcare systems [14].

At the same time, web-native standards became of interest. HL7 FHIR offered a practical synthesis, a synthesis that is simpler to develop than v3’s semantic discipline but remains rigorous enough to be rapidly adopted in EHR systems, clinical research systems, and patient-facing applications, a synthesis that is easy to adopt across all 3 categories of systems and applications and is finding rapid acceptance there [59,85]. In the FHIR, it is reported to be rapidly adopted in the U.S., Europe, and Asia-Pacific, typically as an addition to or replacement of v2 interfaces in chronic disease management systems and national digital-health systems and infrastructures [12].

## The Modern Synthesis: The Architectural Principles and Evolution of FHIR

Healthcare IT environment demanded a standard that would offer the ease of implementation as found in HL7 v2, semantic rigor as found in HL7 v3, and the technological nimbleness of the new web. HL7 came up with FHIR as the solution to this requirement. It is a compilation of the experiences that have been gained out of the past standards, forming a new paradigm of data exchange that is strong and accessible [86].

This part is the evolutionary path of FHIR, including its architectural principles and design philosophy. Its focus is on the RESTful API model that supports web-centric interoperability and the classification, structure, and interlinkage of resources, which are the basic building blocks of the standard [86].

### Learning from the past: The design philosophy and evolutionary path of FHIR

Several studies consistently presents FHIR as a response to many of the implementation and interoperability limitations associated with earlier HL7 standards, particularly the semantic rigidity observed in HL7 v3 environments and the customization burden frequently reported in HL7 v2 deployments [86]. Studies evaluating contemporary interoperability architectures commonly highlight that FHIR adopted widely used web technologies and lightweight exchange mechanisms to simplify implementation and improve accessibility for developers, healthcare institutions, and third-party application ecosystems [86]. A recurring theme across the literature is the emphasis on modular and granular resource-based exchange, which enabled healthcare information to be shared more flexibly than document-centric or monolithic interoperability approaches [86]. Several analyses additionally identify the widely discussed “80/20” design philosophy of FHIR as an important factor supporting adoption because it standardized the majority of common interoperability requirements while still permitting controlled customization for specialized clinical workflows and organizational scenarios [45].

The literature also describes FHIR as being very iterative and implementation-focused, with each successive release being developed based on implementing it in practice, interoperability efforts, and large-scale testing programmes [48]. Early developmental versions introduced lightweight resource-oriented interoperability models appropriate for new mobile and web-based healthcare applications, whereas later versions added more maturity in the development of resources, enhanced API standardization, and boosted implementation stability in enterprise healthcare environments [48]. The Release 4 (R4) has been shown in several studies to be an important milestone as it added normative content to the core resources and increased the level of assurances of long-term support and consistency of implementation [48]. There are multiple literature references that one might find describing the FHIR R4 inclusion in the U.S. ONC 21st Century Cures Act Final Rule as a major regulatory event that helped move the enterprise adoption of FHIR and move on to broader national interoperability efforts [48]. Later investigations also point out that further releases of FHIR continued to build maturity and implementation capability of the resources as the need for healthcare interoperability progressed [48].

Many research works on FHIR governance and standardization highlight the role of FHIR Maturity Model for boosting the implementation confidence and lifecycle management [48]. Unlike previous interoperability standards, which force organizations to implement many parts of the specifications even if they were not production-ready or fully stable, the FHIR Maturity Model’s approach was to apply a staged evaluation to check the stability, production readiness, and implementation maturity of each individual resource [48]. This incremental governance model has been consistently linked to higher trust among developers, greater participation of developers in the ecosystem, and more agile standard evolution in a variety of healthcare interoperability settings [48]. Several analyses additionally suggest that the resource-level maturity framework enabled healthcare organizations to prioritize stable and widely validated interoperability components while continuing to evaluate emerging or experimental resources under controlled deployment conditions [48].

The study also sheds light on the growing influence of FHIR beyond the standard FHIR use cases for interoperability. Boussadi et al. [87] proved that EHR information can be integrated into clinical data warehouse infrastructures, using interoperability approaches that are based on FHIR. It was also reported by other studies that standardized FHIR representations increased FHIR interoperability in healthcare analytics and machine learning workflows by enabling more reuse and semantically consistent FHIR data structures across institutions [88]. Research evaluating public health registries further observed that a substantial proportion of registry data elements could be represented effectively using standard FHIR resources, reinforcing its suitability for scalable public health reporting and surveillance infrastructures [20]. At the same time, scoping reviews related to clinical research environments are increasingly describing FHIR as an important framework for interoperability in the clinical research field for clinical trial management, secondary use of clinical information, and distributed research integration initiatives [89]. Taken together, the literature reviewed describes FHIR as a very flexible framework for interoperability that was equipped with web-native design, modular resources, incremental governance, and an ecosystem-driven evolution that positioned it as a major contributor to the modern digital health integration, scalable analytics, and modern healthcare interoperability infrastructures. The progressive evolution of FHIR releases and their reported interoperability advancements is summarized in Table 2.

**Table 2.** Progressive evolution of FHIR releases and their reported impact on healthcare interoperability adoption. Data synthesized from [48].

Release	Year	Reported interoperability advancements	Adoption/Maturity status
DSTU1	2013	Introduced foundational FHIR concepts emphasizing lightweight mobile applications, document retrieval, and simplified resource-based exchange mechanisms.	Early draft standard for trial use
DSTU2	2015	Expanded implementation adoption, refined extension mechanisms, and accelerated ecosystem participation through initiatives such as the Argonaut Project.	Increasing implementation adoption
STU3	2017	Improved workflow support, enhanced implementation tooling, and strengthened maturity of core interoperability resources.	Standard for trial use
R4	2019	Introduced normative content for core resources including Patient and Observation, while strengthening RESTful API interoperability and enterprise adoption.	Normative content introduced
R5	Ongoing	Expanded normative resources and supported increasingly complex interoperability, analytics, and healthcare integration use cases.	Expanding normative ecosystem

FHIR, Fast Healthcare Interoperability Resources; API, application programming interface; R4, Release 4

### The FHIR RESTful API: A web-centric approach

The main architectural paradigm of FHIR is a Representational State Transfer (REST) API [90]. This is a fundamental breakaway of the stateful, point-to-point messaging of HL7 v2 and rides on the ubiquitous hypertext transfer protocol (HTTP) on which the World Wide Web is built on [86].

The data in a RESTful architecture is represented as a “resource”, which can be accessed by a special URL. Access to such resources is done with the standard HTTP verbs:

• GET: To retrieve (read) or search for resources.
• POST: To create a new resource.
• PUT: To update an existing resource.
• PATCH: To perform a partial update on an existing resource.
• DELETE: To remove a resource [86].

For example, to retrieve a specific patient’s record, a client application would send an HTTP GET request to an end point like {serverURL}/Patient/{id} [86]. This model which is web-centric is stateless by nature that is, every request carries all the information needed by the server to satisfy it. This loosely coupled and decentralized design fits very well into the modern application development and more so in mobile and cloud systems-based applications [90]. This design is both technical- and cultural-based; by using the tools and patterns adopted by the large community of web developers, FHIR has substantially reduced the entrance barrier to health IT innovation, which has played a major role in its rapid and widespread adoption [86].

The use of FHIR is inspired by its API based on REST that is lightweight and easy to use, a standard, compared to Simple Object Access Protocol (SOAP) and proprietary protocols, in line with current web structures [84]. This web-native design reduces the burden of training of the developer and facilitates integration among the EHRs, mobile applications, and Internet of Things medical devices [91]. The statelessness of REST enhances scalability and thus is suitable in cloud-native and distributed health systems [84].

The security is integrated with HTTPS, OAuth 2.0, and SMART on FHIR, which facilitates privacy-preserving data sharing and HIPAA and GDPR compliance [92]. RESTful APIs are also compatible with real-time decision support, predictive analytics, remote monitoring, and distributed systems like federated healthcare systems [11]. FHIR APIs allow access to decentralized data in a standardized manner without fear of confidentiality in federated learning and artificial intelligence (AI)-driven analytics [11].

The practical applications of this effect are highlighted in the following: connecting genomic and clinical data to support precision medicine [84], scaling national COVID-19 reporting systems [91], and enabling interoperable cloud data lakes on Azure and Google Cloud [11]. Altogether, this indicates that REST is not only a transport mechanism but also an essential design concept that makes FHIR the interface of healthcare to the wider digital ecosystem.

This preconditions the analysis of FHIR resources, the modular clinical information units that implement its semantic and structural model.

**Fig. 7.**
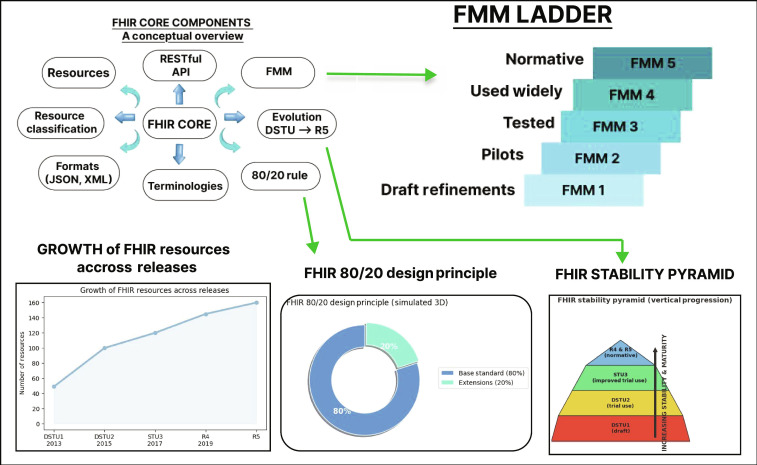
Synthesized overview of Fast Healthcare Interoperability Resources (FHIR) architectural components, resource evolution, maturity progression, design principles, and interoperability stability mechanisms. The visual representations included in this figure reflect qualitative synthesis-based interpretation derived from the reviewed literature and technical documentation. Source: Authors’ synthesis based on reviewed interoperability literature and technical documentation. FMM, FHIR Maturity Model

As illustrated in Fig. 7, FHIR interoperability frameworks combine modular resource-oriented architecture, RESTful communication models, terminology integration, and progressive maturity governance mechanisms intended to support scalable and implementation-oriented healthcare interoperability.

### Resources as the building blocks: Classification, structure, and interlinkage

The basic conceptual and data-level unit in FHIR is the Resource [13]. A Resource is a modular, self-contained packet of clinical or administrative information. Each resource type (e.g., Patient, Condition, MedicationRequest, and Encounter) has a defined identity, a structured data content with required and optional elements, and a common set of metadata [93]. As of FHIR R4, there are 145 defined resources [48]. These resources are represented using modern, easily parsable data formats, primarily JSON and XML, with support for Resource Description Framework as well [86].

To handle this huge amount of resources and to comprehend the relations between them, the FHIR specification divides them into a logical classification system with multiple layers or modules [13]. This structure is used to explain the architecture and dependencies of the standard. The resources are loosely categorized into functional domains like administrative (e.g., Patient, Practitioner, and Organization), clinical (e.g., Condition, Observation, and Procedure), financial (e.g., Claim and Invoice), infrastructural (e.g., Bundle and Binary), and conformance-related (e.g., CapabilityStatement and StructureDefinition) [48,93]. This modular categorization facilitates partitioning of concerns and makes it easier to implement it in various healthcare systems.

An important architectural trait is the fact that resources are not monolithic but they are interconnected via references. A blood pressure Observation resource of a patient, for example, will not have the complete demographic data of the patient but will include a reference (a URL) to the actual Patient resource to which it is applied. This referential design has been created as a modular design that does not duplicate data and that can be structured into a distributed, web-like network of clinical information [94] where consistency is delivered by sharing a set of common elements like “id”, “meta”, and “extension” that gives identity, versioning, and extensibility throughout the ecosystem [13,93]. The extensions and profiles enable implementers to customize the base resources to fit their use cases while remaining interoperable, which was the flaw affecting the adoption of HL7 v2 and CDA because of the variability of resource usage [95]. This multifaceted solution renders the resources standardized and flexible to ensure the data exchange can be scaled across various clinical processes in a standardized manner [96]. The major categories of FHIR resources and their interoperability roles are summarized in Table 3.

**Table 3.** High-Level classification of FHIR resources and their interoperability roles. Data synthesized from [13].

Level	Interoperability role	Representative resources
Foundation	Defines core infrastructure, terminology, and conformance elements supporting interoperability across FHIR implementations.	StructureDefinition, CapabilityStatement, CodeSystem, ValueSet, Bundle, OperationOutcome
Base	Represents foundational healthcare entities frequently referenced across interoperability workflows and clinical exchange processes.	Patient, Practitioner, Organization, Location, Device, Encounter, Group
Clinical	Supports exchange of core clinical information generated during patient care and healthcare delivery workflows.	Observation, Condition, Procedure, MedicationRequest, MedicationAdministration, AllergyIntolerance, DiagnosticReport, CarePlan
Financial	Supports interoperability of billing, reimbursement, coverage, and healthcare financial management processes.	Coverage, Claim, ExplanationOfBenefit, Invoice, Account
Specialized	Supports advanced interoperability use cases including public health, research, genomics, and evidence-based healthcare applications.	ResearchStudy, Measure, MeasureReport, Questionnaire, PlanDefinition, GenomicStudy

FHIR, Fast Healthcare Interoperability Resources

### FHIR resource versioning and backward compatibility

FHIR is devised in such a manner that it can be extended without breaking current deployments and thus backward compatibility as well as versioning are the 2 principles of sustainable interoperability [97]. Resources also move through specified levels of maturity, namely, Draft, Trial Use, and Normative, and a rule is that once a resource is Normative, all subsequent revisions must be backward compatible [92]. Non-normative content can still undergo structural or binding changes between releases, which can sometimes complicate long-term stability [98]. In the implementation layer, the resources have an intrinsic version metadata (meta.versionId, lastUpdated), allowing the retrieval of the whole history and conditional updates based on the ETags to manage the concurrency [92,97]. Audit trails, rollback, and regulatory compliance are supported with the help of HTTP controls, including If-Match and If-None-Match, which enforce optimistic concurrency [98].

Maintaining backward compatibility across major FHIR releases remains difficult. Although HL7 prioritizes additive, nonbreaking updates, some structural changes are unavoidable [6]. In response, many production servers (e.g., Smile CDR) often have support for multiple versions of FHIR together or middleware to mediate between versions so that they can be progressively adopted [99]. However, the practical examples have version divergence problems with structures, terminology bindings, and IG conformance impeding smooth exchange [100]. Best practices therefore emphasize defensive client design, adherence to profiles and conformance resources, and strong governance and tooling ecosystems as essential factors for ensuring consistent, safe, and interoperable data exchange even within FHIR’s robust versioning model [97,100].

### Security and access control in FHIR

Security and access control are complementary layers built on the premise of versioning and backward compatibility to provide the safety of sensitive health data handling. FHIR provides a framework for implementing resource-level authentication and authorization, enabling fine-grained control over who can access, create, or modify resources through implementation-specific security mechanisms [6,92]. OAuth 2.0 is a protocol used as a standard of delegated authorization, which allows third-party applications to gain access to patient data safely without involving credentials exchange [92]. SMART on FHIR adds to them with user consent, patient-centric access control, and contextual permissions that allow safe and standard interactions across different clinical systems [6,99]. Together, these mechanisms support regulatory compliance (e.g., HIPAA and GDPR), real-time auditability, and secure interoperability between systems [6,92,98–100].

Given the sensitive nature of healthcare data and the diverse sources and formats of information that ensures consistent interpretation and semantic clarity is equally critical. This underscores the need for standardized terminologies and code systems that define shared vocabularies and enable accurate communication across systems. Consequently, security mechanisms and controlled access must work hand in hand with standardized terminologies to maintain both the safety and semantic integrity of health data, naturally leading to the discussion of terminologies and code systems in FHIR resources in the next subsection.

### Terminologies and code systems in resources

To ensure that the data is understandable and meaningful across different health systems and can be exchanged consistently, FHIR resources make use of standard terminologies and code systems. Many resources make common usage of the common terminologies like SNOMED CT for clinical concepts, LOINC for laboratory observations, and ICD-10 for diagnosis to ensure consistency and transparency in the interpretation of data when shared across applications and institutions. Such terminologies enable machines and humans to process and understand healthcare data in a similar manner, facilitating large-scale analytics, clinical decision-making, and regulatory reporting processes [98,99].

The coded elements in FHIR resources are often linked to terminology bindings, ValueSet constraints, and standardized system Uniform Resource Identifiers to enable semantic validation and interoperable exchange of data in diverse healthcare contexts. This explicit terminology integration allows software systems to check for consistency of exchanged information, with standardized vocabularies, and help in reducing semantic ambiguity and overall data incoherence [97,100]. FHIR additionally supports concept maps that facilitate interoperability across multiple coding systems and institutional environments where different terminology standards may coexist simultaneously [92,99].

The use of standardized terminologies in FHIR profiles and resource definitions further enhances the semantic interoperability, thereby also contributing to regulatory compliance, interinstitutional interoperability, and global healthcare data exchange. These terminology-governance mechanisms are also illustrative of some key semantic interoperability principles that have been adopted from HL7 v3 environments and adapted into modern FHIR-based interoperability architectures [6,92,97–100].

## Adapting FHIR to the real world: Profiles, extensions, and IGs

The basic FHIR specification offers a universal starting point to healthcare data exchange, yet there is operational interoperability in contexts of jurisdiction, organization, and clinical. This is made possible by the conformance layer of FHIR-Profiles, extensions, and IGs, which provides a formal, computable model of controlled customization rather than the uncontrolled diversity of HL7 v2 and the unduly rigid HL7 v3 [36,39]. Profiles can restrict or elaborate the resource capacity to meet national and organizational requirements, including the case of the US Core and the UK Core IGs, where the resources ensure alignment of regulations with cross-border interoperability [4,30,35]; extensions enable additional attributes while preserving semantic integrity, preventing ad hoc customization [33]; and IGs consolidate constraints into enforceable rules, validation artifacts, and examples that promote consistent adoption across vendors and healthcare systems [25,26,38]. Although local adaptations risk fragmentation, HL7’s FHIR Registry and international harmonization efforts mitigate divergence by encouraging reuse and alignment [27,32], collectively anchoring FHIR’s flexibility within a governed framework and enabling meaningful comparison with earlier interoperability standards.

### Constraining and extending the standard: The role of profiles

A FHIR Profile is a collection of rules that alters a FHIR base resource to fit a specific situation [13]. The main mechanism used in the enforcement of particular data requirements and business rules over the generic base specifications are profiles. They are not separate resource types but are instead defined using a foundational FHIR resource called StructureDefinition [101].

A profile can apply several types of modifications to a base resource:

• Constraining Cardinality: An element that is optional in the base resource (e.g., cardinality of 0..1) can be made mandatory in a profile (cardinality of 1..1) [102]. For instance, the US Core Patient Profile mandates that a patient must have at least 1 identifier [102].
• Binding to Terminology: A coded element can be “bound” to a specific ValueSet, which is a defined list of permissible codes from 1 or more code systems (like LOINC or SNOMED CT). This ensures that data is coded consistently [101].
• Restricting Data Types: For elements that can hold multiple data types (e.g., value[x]), a profile can restrict it to a single type (e.g., valueQuantity).
• Adding Extensions: As detailed below, profiles can declare that specific extensions must be used to carry information not present in the base resource [102].

This capability of generating formal, machine-computable definitions of context-specific requirements is central to attaining semantic interoperability. A StructureDefinition can be programmatically accessed through systems to be used and to ensure that a particular resource instance is as per the rules of a particular profile [95].

Practically, national and global initiatives like US Core, UK Core, and the International Patient Summary can demonstrate how profiles are implemented in order to impose regulatory requirements and do standardized information exchange across jurisdictions [4,30,35]. HL7 and community project validation tools are also used to supplement their role, enabling quality and consistency of automated conformance checks throughout implementation [26,32]. However, similarly, the increase in local profiles increases the chances of interoperability fragmentation, and, in this respect, global registries and coordinated governance mechanisms are necessary [27].

Profiles are therefore the tools to bring into reality the abstract flexibility of FHIR in interoperable and enforceable rules. By constraining optionality, aligning with standardized terminologies, and integrating structured extensions, they ensure that diverse implementations remain comparable and verifiable across healthcare ecosystems [13,95,101,102]. Simultaneously, there are the situations when absolutely new data items must be added in order to reflect the information that is not included in the base resources, making one think of the mechanisms that are dedicated to the latter.

### Adding new data elements: Extensions and modifier extensions

An extension may be added to a use case when the use case needs a data element that the underlying FHIR resource does not support [102]. It is a critical characteristic that enables the standard to be modified without modifying the fundamental specifications, which means that it is backward-compatible. An extension is a key–value pair that is structured:

• The key is a canonical URL that uniquely identifies the extension’s definition. This URL should point to a StructureDefinition that formally defines the extension’s purpose, data type, and rules [103].
• The value can be any valid FHIR data type, from a simple primitive (valueString, valueBoolean) to a complex CodeableConcept or even nested extensions [103].

For example, the base Patient resource does not include an element for race. The US Core Patient Profile, in turn, proposes this notion in the form of a formally defined extension (http://hl7.org/fhir/us/core/StructureDefinition/us-core-race), which allows representing race information in a standardized format, without making changes to the core resource [102]. Information systems that are aware of this extension are able to properly understand and act on it, and information systems that are not aware of it can safely ignore the element without affecting the interoperability. This practice is an illustration of the FHIR concept of controlled flexibility, whereby it can be customized without breaking baseline compatibility between heterogeneous systems.

Another special and safety-critical category of this mechanism is the modifier extension. Modifier extensions are applied in the case where the extra information has a fundamental change to the clinical meaning of the element to which it is attached. As an example, a modifier extension on a MedicationRequest that states that the medication should not be administered cannot be disregarded without danger. The systems that face an unknown modifier extension thus need to decline the resource or raise a clear warning because processing the resource before the knowledge of the modifier may jeopardize patient safety [103]. Extensions and modifier extensions are normally administered using profiles, which stipulate their application guidelines, compulsory versus elective status, and value limits. Together, these mechanisms allow FHIR to safely incorporate new and safety-critical data elements, supporting national frameworks (e.g., US Core and UK Core) and local requirements while maintaining semantic integrity and interoperability [102,103]. This systematic management of extensions is a direct pass to the concept of Mandatory and Must Support elements that determine how systems should implement and process constrained data elements in profiles.

**Fig. 8.**
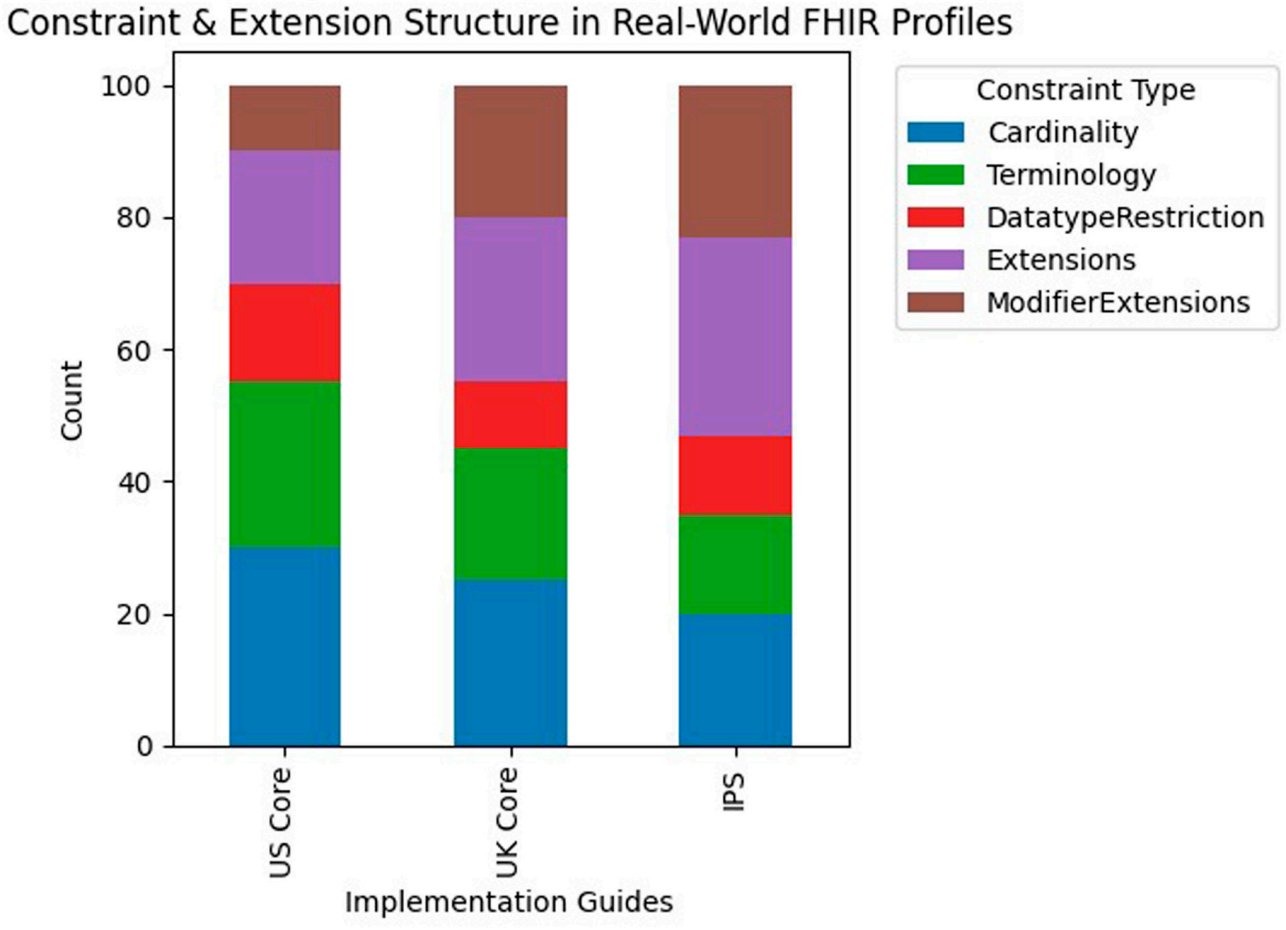
Comparative distribution of constraint and extension patterns across selected Fast Healthcare Interoperability Resources (FHIR) implementation guides. Source: Authors’ synthesis based on reviewed interoperability literature and technical documentation.

As illustrated in Fig. 8, real-world FHIR IGs frequently apply varying combinations of cardinality constraints, terminology bindings, datatype restrictions, and extension mechanisms to support institution-specific interoperability requirements.

### The “Must Support” vs. “Mandatory” distinction

Two important terms for conformance of elements are presented in Profiles that demonstrate a profound, practical knowledge of the real healthcare data environment: “Mandatory” and “Must Support” [102].

• Mandatory: This is a cardinality constraint. If an element in a profile has a minimum cardinality of 1 (e.g., 1..1 or 1..*), it is mandatory. This means that every instance of a resource conforming to that profile must contain a value for that element. The absence of the element makes the resource nonconformant [102].
• Must Support: This is a flag that applies to an element. It does not mean the element must always be present. Instead, it means that a system claiming conformance to the profile must be capable of processing, storing, and making the element available if it is present in an instance it receives. The system cannot simply ignore the data [102]. When an element is expected but its value is unavailable, FHIR also provides the Data Absent Reason extension to explicitly communicate why the data are missing, thereby preserving semantic clarity and interoperability [102].

This difference is delicate but essential since it distinguishes between a need to be proficient in handling data and a need to always do so. This design is a direct reflection of the clinical reality, in which some information may be unavailable at certain stages of care. It is possible thus that a system will still be conformant even though it is unable to provide a Must Support element but nonconformant when it cannot correctly interpret that element when received. This model separates availability and capability and thereby maintains semantic interoperability and makes conformance achievable.

Must Support designation is particularly valuable in nonhomogeneous settings of healthcare where data availability and workflows differ in many ways between systems. Profiles minimize semantic ambiguity and curb clinical misinterpretation or workflow failures by indicating what elements should be properly processed in case they appear in a given profile [102,103]. This difference is operationalized within the validation settings: Mandatory elements are strictly adhered to during the process of the instance validation, and Must Support elements are checked with the help of the capability assertions, that is, systems can safely consume optional but clinically significant information [95]. This balance does not provide the extreme rigidity and yet promotes the trustful interoperability.

The Must Support concept also directly compliments the usage of Extensions and modifier extensions. To ensure semantic consistency among implementations, it is important to mark critical information when communicated using extensions as Must Support, such that receiving systems are aware of their value and properly handle it accordingly [103]. From an implementation perspective, this mechanism further guides development priorities: Mandatory elements define the minimal interoperable baseline, while Must Support elements direct incremental enhancements that improve clinical completeness without jeopardizing conformance [102].

Based on these conformance principles at the element level, profiles should also be packaged, governed, and interpreted uniformly across deployments. IGs are thus the official mechanism by which profiles, extensions, and conformance rules are implemented, thereby ensuring reproducibility and alignment across interorganizational and interjurisdictional settings. The next subsection discusses the implementation of Scalable and Reliable FHIR-based interoperability through IGs.

### Packaging conformance: The role of IGs

While profiles and extensions define individual rules, an IG packages them together into a cohesive, publishable, and implementable specification for a complete use case or jurisdiction [45]. IGs serve as the practical bridge between the abstract FHIR specification and real-world deployment, ensuring that all conformance rules—including profiles, extensions, “Must Support” elements, mandatory fields, and terminology bindings—are presented in a structured and machine-readable format that can be consistently implemented across diverse systems [92,97]. An IG is a collection of FHIR conformance resources—including StructureDefinitions (for profiles and extensions), ValueSets, CodeSystems, ConceptMaps, and CapabilityStatements—along with human-readable narrative documentation that explains how to use them together [45].

Prominent examples of IGs include:

• The US Core IG: Defines the base profiles, extensions, and terminologies that all systems must support to meet U.S. regulatory requirements for patient data access and interoperability [104]. This IG operationalizes constraints described in the “Constraining and extending the standard: The role of profiles” section, integrates extensions as described in the “Adding new data elements: Extensions and modifier extensions” section, and clearly defines which elements are mandatory versus “Must Support” as explained in the “The ‘Must Support’ vs. ‘Mandatory’ distinction” section, enabling uniform implementation and validation across healthcare systems [98].
• The SMART App Launch Framework IG: Defines the profiles and operations needed to support the SMART on FHIR secure application launch protocol [105]. It demonstrates how IGs can include workflow, security, and operational requirements alongside data structure specifications, ensuring that applications interact safely and predictably within the FHIR ecosystem [6].
• The Genomics Reporting IG: Specifies the profiles and patterns for representing and exchanging complex genomic data using FHIR resources [106]. This IG exemplifies how IGs can accommodate highly specialized domains, allowing extensions and modifier extensions to capture nuanced data without compromising the core standard, maintaining backward compatibility, and supporting semantic interoperability [99].

IGs are essential for translating FHIR’s flexible specification into enforceable, context-specific rules. They formalize constraints on cardinality, data types, and terminology bindings, while supplying the narrative and metadata required for implementers to interpret “Must Support” elements, manage modifier extensions safely, and incorporate custom extensions without violating the base specification [97,100].

IGs additionally make automated conformance validation possible so that systems can test resource instances with published profiles, extensions, and value-set bindings. This ensures semantic consistency, reduces implementation variability, and supports interoperability across heterogeneous clinical, regional, and organizational environments [6,92].

Serving as the operational pathway for applying FHIR, IGs bundle profiles, extensions, “Must Support” designations, and associated metadata into coherent, testable specifications. In doing so, they convert the theoretical goals of FHIR—flexibility, semantic interoperability, and backward compatibility—into executable implementation frameworks deployed in real systems [98,99]. Their structured packaging of conformance rules provides a scalable framework for cross-jurisdictional and domain-specific adoption, reducing ambiguity, implementation drift, and unsafe data handling [97,100]. This foundation enables the transition to a broader comparative view of FHIR within the continuum of interoperability standards.

## A Comparative Technical Analysis of Interoperability Standards

It is not a straight line of progress of HL7 v2 to HL7 v3/CDA and then to FHIR but a process of action–reaction determined by changing technological constraints and interoperability priorities. HL7 v2 was successful due to simplicity, even though there was no formal semantics, but HL7 v3/CDA was rigorously modeled and was difficult to apply in practice in reality [47,97,98]. FHIR summarizes these lessons as combining flexibility with formal structure and web-native design, in a worldwide trend of shifting from tightly coupled protocols to lightweight APIs [92,99,100]. Such a path offers the basis of comparative analysis of the standards between the modeling, architecture, complexity, and extensiveness to know why FHIR has become the most popular framework.

### Data modeling and granularity

• HL7 v2: Has an implicit, segment-based data model. The basic unit of exchange is the whole message. To retrieve 1 piece of data, one had to process the whole message [47], which made it easy to implement but also caused variance, as frequently there was no common semantic model and thus they could not be implemented uniformly across different institutions [97]. The message-based structure made it hard to have access to or reuse individual data items without retrieving the whole structure and imposed scaling limits and hindered interoperability at a fine-grained level in the message structure [92].
• HL7 v3/CDA: Uses a formal, universal object model—the RIM. In the case of CDA, the unit of exchange is the whole clinical document. It has an internally structured but monolithic object when viewed through the exchange perspective [47]. This change to a semantically rigorous base was a major improvement over HL7 v2 because it entailed a meaning carried into the data model itself [99]. Nevertheless, the RIM was frequently not flexible, and document-centric CDA was not well suited to exchanging a whole document to retrieve or modify a single data item in dynamic clinical workflows, which was inefficient in real clinical settings [100]. Consequently, CDA tended to be semantically consistent but not practical and usable in practice [98].
• FHIR: Uses a resource, modular, data model. The resource is the unit of exchange. This gives it a high level of granularity, allowing applications to request and interact with just the individual data they require [86], FHIR also supports modern applications, including mobile health apps, clinical decision support, and patient-facing portals by providing individual, reusable resources such as Patient, Observation, and MedicationRequest [92]. The granularity of resources not only reduces overhead but also enhances semantic interoperability when combined with profiles and extensions [6]. Moreover, the resource-based model aligns with RESTful principles, allowing partial retrievals, updates, and efficient querying that were difficult under prior standards [97,100]. This modularity is widely recognized as one of the core innovations that distinguishes FHIR from its predecessors and underpins its growing global adoption.

As a whole, this development of a segment-based message model of HL7 v2 to a monolithic document model of HL7 v3/CDA to the more granular, reusable, and efficient resource-based data model of FHIR seems to be evidence of a definite direction toward a more refined level of granularity, reusability, and more effective access to data. This development allows applications to request only the data that they require making them less complex, enabling real-time interoperability, and allowing integration among heterogeneous healthcare systems and overcoming the limitations of previous models [97–99].

### Architectural and technological underpinnings

• HL7 v2: It is an event-based point to point messaging architecture. It is based on a stateful, specialized network protocol, the MLLP, which uses Transmission Control Protocol/Internet Protocol. This architecture was a text format, custom and pipe-delimited, called ER7 [44], and it enabled messaging that was low-latency and real-time and was adopted swiftly in hospitals. However, the point to point design brought tight bonding to the systems that made scale and integrating composite systems difficult. The custom ER7 format needed bespoke custom parsers, thereby constraining intervendor compatibility, as well as adding implementation variability to the mix [92,97].
• HL7 v3/CDA: It has a document-oriented architecture. It uses a strictly defined XML data format. Exchange can be done as simple file transfers [47], HL7 v3/CDA enhanced semantic clarity and consistency by using a document-based approach. XML provided rigid validation and hierarchical presentation of complicated clinical data. However, the document-based model was relatively rigid and was often less effective for real-time clinical workflows, as accessing or updating a specific piece of information required processing the document as a whole. The fact that it relies on XML schemas and can sometimes be bulky transport mechanisms discouraged the use in dynamic healthcare setting [98,99].
• FHIR: Its main architecture is a RESTful API service. It utilizes standard stateless web protocols (HTTP/S) and current data formats (primarily JSON and XML) [86], to overcome the limitation of its predecessors and support the modular, granular, and real-time access to clinical data. Stateless RESTful services make integration easy, minimize coupling, and can be used extensively with common web infrastructure. JSON support enables mobile and web applications to exchange data in a lightweight manner, whereas XML maintains compatibility with the legacy systems. FHIR also has added other transport models like messaging, GraphQL, and subscription model, which can be used in both synchronous and asynchronous workflow with supported flexibility [6,100]. Moreover, this architecture is scalable by nature, cloud deployment, and current application paradigms that comply with interoperability standards with modern software engineering practices [97,100].

Altogether, the architectural evolution of HL7 v2 event-driven messaging into v3/CDA document-orientation and, finally, FHIR RESTful and modular API can be seen as the planned transition into being more scalable, more flexible, and more interoperable by design both in terms of technology and clinical requirements with the minimal trade-offs that earlier standards have demonstrated to impose on them [6,92].

### Implementation complexity and developer experience

• HL7 v2: It requires a thorough understanding of HL7 message structures and the MLLP protocol for implementation. The developer experience may be challenging when one is not immersed in traditional health IT experience [47], since HL7 v2 is widely used and mature but lacks formal tools to verify compliance and may vary across vendor implementations leading to inconsistencies and time wasting troubleshooting. Messages have to be manually parsable, and message edge cases need to be addressed by the developers, potentially hindering quick development and innovation [92,97].
• HL7 v3/CDA: Implementation is widely regarded to be the most complex. It requires profound knowledge of the abstract RIM and complicated XML schemas. The learning curve is steep [47]. The HL7 v3/CDA is semantically rigorous but at the cost of accessibility due to its formal structure. Implementing compliant systems may require specialized training, a lot of reference material, and strong tooling on the part of the developers. This complexity may slow down the project schedule and necessitates greater investment of resources rendering it less viable when one is working with a smaller organization or in cases of quick deployment of the project [98,99].
• FHIR: It is much easier to implement. FHIR utilizes established skills and vast ecosystem of tools used by the global web developer community, that the barrier to entry is greatly reduced by using REST, HTTP, and JSON [86]. FHIR also has a wide array of open-source libraries, validation tools, and community support that simplify the implementation and testing process. The modular design enables incremental adoption where developers can implement only the resources or profiles that they need to use in their case. Also, the fact that FHIR can integrate with the latest development workflows such as agile development, continuous integration, and cloud-native deployment makes developers more productive and can reduce the time to market faster [6,100]. This usability has helped it achieve immediate acceptance both in clinics and research settings to bridge the gap between health IT professionals and the general web developers [97,100].

All of these differences together highlight the fact that the development of HL7 v2 to v3/CDA and its successor, FHIR, not only mitigated the technical aspects of interoperability but also developed a strategic approach to reducing the barriers to implementation and making contemporary healthcare data exchange more accessible, reliable, and easier to develop.

### Extensibility and conformance

• HL7 v2: Informal “Z-segments” are commonly used to deal with extensibility. Conformance is handled through text-based IGs, which tend to be ambiguous and not machine-readable [52]. Although HL7 v2 Z-segments have certain flexibility, they are mostly ad hoc resulting in the inconsistencies across implementations. No standardized methodology exists to formally validate such extensions and share them that can lead to interoperability problems when various vendors or institutions seek to exchange data [92,97]. Also, conformance guides are text-based and, as a result, automated conformance checking is constrained, meaning that a manual review and interpretation are necessary [98].
• HL7 v3/CDA: Extensibility can exist but limited by the rigid RIM. The conformance is ensured by templated XML schemas, which are formal and difficult to develop [65], but the steep learning curve and the sheer complexity ensure that the practical adoption is not easily possible with HL7 v3/CDA. Although XML templates provide consistency, they can be costly in terms of tooling and specialized expertise. Local extensions or modifications may be difficult to implement and transferring such extensions across organizations may prove to be burdensome [6,99].
• FHIR: Extensibility is a core, formalized feature managed through computable StructureDefinition resources. Conformance artifacts are themselves FHIR resources, providing a “best of both worlds” approach: controlled, standardized flexibility [101]. FHIR’s approach enables both rigorous semantic interoperability and practical adaptability. Extensions are formally defined and machine-readable and can be shared across systems without altering the base resource. Profiles, IGs, and computable conformance artifacts allow organizations to enforce context-specific rules while still adhering to a global standard. This controlled flexibility ensures that developers can meet local or jurisdictional requirements without fragmenting the standard, substantially reducing the risk of interoperability failures [100,107]. By integrating conformance and extensibility into the resource framework itself, FHIR addresses both the semantic rigor of HL7 v3 and the practical flexibility that was lacking in HL7 v2 [97,100].

**Fig. 9.**
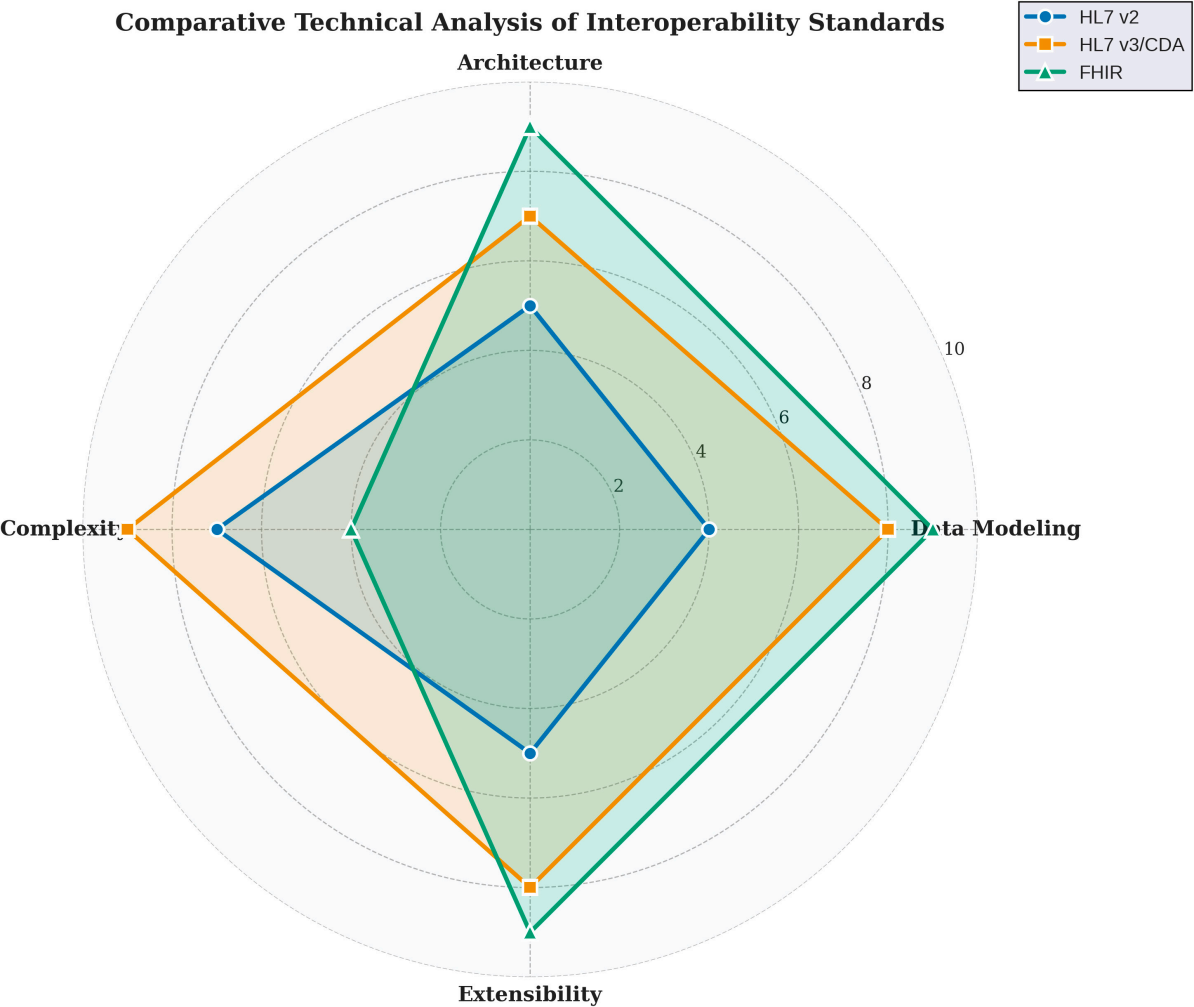
Qualitative comparative synthesis of HL7 v2, HL7 v3/Clinical Document Architecture (CDA), and Fast Healthcare Interoperability Resources (FHIR) across selected interoperability and architectural dimensions. The comparative scoring shown in this figure reflects synthesis-based interpretive analysis derived from the reviewed literature rather than standardized quantitative benchmarking measurements. Source: Authors’ synthesis based on reviewed interoperability literature and technical documentation.

Figure 9 presents a comparative qualitative synthesis of major architectural and interoperability characteristics associated with HL7 v2, HL7 v3/CDA, and FHIR frameworks based on recurring themes identified throughout the reviewed literature.

To recap it all, the history of the developments of HL7 v2 through HL7 v3/CDA to FHIR can be seen as the gradual move toward more formal and semantically consistent and implementation-friendly interoperability frameworks. This development highlights the long-term struggles that have long defined the evolution of healthcare data standards, as well as the technical maturation of them. These findings precondition the ultimate findings of this review, which generalizes the overall implications of this development on the future of interoperable digital health systems.

## Results and Qualitative Synthesis

### Descriptive trends in the literature

The literature reviewed reveals that there are clear evolutionary stages in the history of the development of healthcare interoperability standards, with early attempts to standardize the interfaces between fragmented institutions of communication (HL7) moving on to semantically consistent and web-oriented interoperability frameworks. The literature shows that there has been a substantial acceleration of policies in recent years, such as the widespread adoption of EHRs, Meaningful Use requirements, and the greater focus on semantic interoperability in heterogeneous healthcare systems. The studies included are publications from major biomedical and health informatics journals (e.g., JAMIA and JMIR), technical documentation published by HL7 International, technical guidance documents that are implementation-oriented, and interoperability guidance documents from regulatory bodies such as the ONC. Taken together, the literature outlines the shift from a segment-based HL7 v2 message-based approach to implementation of a document-centric HL7 v3/CDA architecture, and, in recent years, the move toward resource-oriented FHIR interoperability approaches. Looking at general publication trends, there is growing maturity in technology, regulatory momentum, and greater academic consensus that FHIR is a well-founded, modern framework for interoperability that is widely adopted across healthcare systems, coexisting with legacy interoperability infrastructures in hybrid healthcare systems.

### Thematic scope of included studies

The included studies encompass a broad yet interconnected range of themes related to healthcare interoperability research. This encompasses the design, implementation, and evaluation of applicable interoperability architectures based on HL7, CDA, and FHIR, as well as the integration of EHRs in a variety of healthcare settings, semantic and syntactic harmonization of clinical data, and web-native, API-driven models for scalable healthcare information exchange. Other themes include governance of interoperability, regulatory compliance, conformance validation, privacy protection, data provenance, and operational integration of interoperability frameworks in clinical workflows. The literature also indicates the rising research interest on the combination of interoperability standards and AI applications, applied to clinical decision support systems, and to large-scale health information exchange infrastructures. These thematic domains are analyzed as a whole, with the results reported on in the comparative thematic synthesis below.

### Comparative thematic synthesis

The reviewed studies as a whole have shown the evolution of standards for healthcare interoperability from HL7 v2 to HL7 v3/CDA and then to FHIR as a progressive move to overcome the structural, semantic, and implementation problems that arose with previous interoperability architectures. HL7 v2 has been generally recognized as a widely used and flexible interoperability framework, but often, there was a lack of semantic consistency between different healthcare environments, due to extensive local customization and implementation variability. HL7 v3 and CDA sought to overcome these drawbacks with formally structured and semantically consistent models based on the RIM, but challenges with implementation complexity, adoption barriers, and operational overhead remain prominent in the reviewed literature as important factors to prevent broader implementation.

The included literature further characterizes FHIR as an interoperability framework that integrates the implementation-oriented strengths of HL7 v2 with the semantic objectives of HL7 v3 while simultaneously leveraging modern web technologies and resource-oriented interoperability mechanisms. In all the studies reviewed, FHIR was found to be consistently linked to greater scalability, ease of developer access, API-focused integration, and enhanced support for new digital health ecosystems. Concurrently, the literature highlights that legacy HL7 v2 and CDA infrastructures are expected to continue to exist within healthcare systems due to regulatory requirements, institutional investments, and established clinical workflows. The overall picture of these thematic findings is that interoperability is an ongoing capability that has been influenced by technology, governance, implementation practices, and the growing complexity of the healthcare data landscape. A comparative summary of the technical characteristics, interoperability mechanisms, strengths, limitations, and implementation considerations of HL7 v2, HL7 v3/CDA, and FHIR is presented in Table 4.

**Table 4.** Comparative analysis of HL7v2, HL7v3/CDA, and FHIR. Data synthesized from [47].

Comparison dimension	HL7 v2.x	HL7 v3 / CDA	FHIR
Paradigm	Event-driven, point-to-point messaging	Model-driven; document-oriented (CDA)	Resource-oriented, RESTful API services
Data model	Implicit, segment-based	Universal, object-oriented (RIM)	Modular, granular resources
Data format	ER7 (pipe-and-hat delimited text)	XML	JSON, XML, RDF
ff Transport protocol	MLLP over TCP/IP	Various (HTTP, SMTP, etc.)	HTTP/S
Granularity	Message-level	Document-level	Resource-level
Architecture	Tightly coupled, stateful connections	Loosely coupled	Loosely coupled, stateless
Extensibility	Informal (Z-segments)	Formal but rigid (constrained by RIM)	Formal and flexible (Profiles, Extensions)
Implementation complexity	Moderate; requires specialized knowledge	Very high; requires deep RIM/XML expertise	Low; uses standard web technologies
Tooling	Specialized integration engines	XML-focused tools	Standard web development libraries/frameworks
Primary use case	Legacy system integration (laboratory, ADT)	Clinical document exchange (CDA)	Web/mobile apps, modern system integration

CDA, Clinical Document Architecture; FHIR, Fast Healthcare Interoperability Resources; API, application programming interface; ADT, admission–discharge–transfer; JSON, JavaScript Object Notation; RDF, Resource Description Framework; MLLP, Minimal Lower Layer Protocol; RIM, reference information model; XML, Extensible Markup Language; HTTP, hypertext transfer protocol; TCP, Transmission Control Protocol; IP, Internet Protocol; SMTP, Simple Mail Transfer Protocol

## Synthesis, Limitations, and Future Directions

The reviewed literature shows that the transition from HL7 v2 to HL7 v3/CDA and finally to FHIR has been a gradual process toward improving the semantic interoperability of health information, structured data representation, and web-based integration of health information, but each standard has its own set of practical limitations. HL7 v2 remains extensively embedded within healthcare infrastructures because of its implementation flexibility, although studies consistently report interoperability inconsistencies arising from localized customization and heterogeneous interface implementations. HL7 v3/CDA provided more sophisticated semantic modeling and formalized clinical meaning, but some implementations were complex and were met with major barriers to adoption. A number of these limitations were addressed by FHIR in its modular resources, RESTful architectures, and developer-oriented interoperability approaches, but it is still noted that there are challenges with profiling variability, uneven resource maturity, and integration with legacy systems and historical data models.

Several methodological limitations need to be taken into consideration when interpreting the findings of this review. While a systematic search was conducted in the biomedical and technical databases, this search may not have been exhaustive and studies indexed in other repositories might not have been captured. The addition of gray literature and technical documentation that are implementation-oriented can also result in reporting differences in quality and methodological consistency between sources. Moreover, as the comparative thematic synthesis was based on interpretative analysis of diverse technical literature, selection and extraction bias may have been present even though the procedures of eligibility and extraction used were predefined.

The scope of this review is also restricted to English literature and HL7-family standards, with comparatively little consideration of complementary interoperability frameworks like DICOM and openEHR. Across the included studies, future research directions consistently emphasize stronger conformance governance, more reliable migration strategies between legacy and modern interoperability environments, and broader cross-standard harmonization capable of supporting scalable analytics, AI-enabled healthcare applications, and high-fidelity clinical data exchange across real-world healthcare ecosystems.

## Conclusion

This structured scoping review demonstrates that the evolution of healthcare interoperability standards from HL7 v2 through HL7 v3/CDA to FHIR reflects a progressive effort to improve semantic consistency, interoperability scalability, and implementation flexibility across increasingly complex healthcare ecosystems. The literature indicates that while HL7 v2 established the operational foundation for large-scale healthcare messaging, subsequent standards addressed limitations related to semantic interoperability, architectural rigidity, and cross-system integration through increasingly structured and web-oriented interoperability models. FHIR currently represents one of the most implementation-oriented and adaptable interoperability frameworks among contemporary standards; however, the continued coexistence of legacy HL7 infrastructures highlights the ongoing need for stronger conformance governance, cross-standard harmonization, and sustainable migration strategies capable of supporting semantically consistent and scalable healthcare data exchange across heterogeneous digital health environments.

## Ethical Approval

Not applicable.
