# Supplementary material for: Deconstructing the Interoperability Stack: A Comparative Technical Analysis of HL7 v2, CDA, and the FHIR Resource Framework
Source: Comput Struct Biotechnol J. 2026 Aug 5;35(1):0168. doi: 10.34133/csbj.0168 (PMC13438035; doi:10.34133/csbj.0168)
Supplement: Supplementary file 1 [file csbj.0168.f1.docx]

**Supplementary Material**

Supplementary Table S1. Database-Specific Search Strategies

| Database | Search Strategy |
| --- | --- |
| PubMed/MEDLINE | ("HL7 v2" OR "HL7 version 2" OR "HL7 v3" OR "Clinical Document Architecture" OR "CDA" OR "FHIR") AND ("interoperability" OR "semantic interoperability" OR "health information exchange") AND ("electronic health records" OR "EHR" OR "health information systems") |
| IEEE Xplore | ("HL7 v2" OR "HL7 v3" OR "FHIR" OR "Clinical Document Architecture") AND ("interoperability" OR "semantic interoperability" OR "healthcare interoperability") AND ("electronic health records" OR "EHR") |
| ScienceDirect | ("HL7" OR "FHIR" OR "CDA") AND ("healthcare interoperability" OR "semantic interoperability" OR "health information exchange") AND ("electronic health records" OR "health information systems") |
| SpringerLink | ("HL7 v2" OR "HL7 v3/CDA" OR "FHIR") AND ("interoperability" OR "health information exchange") AND ("electronic health records" OR "EHR") |
| JMIR | ("FHIR" OR "HL7" OR "Clinical Document Architecture") AND ("interoperability" OR "semantic interoperability") AND ("electronic health records" OR "EHR") |
| JAMIA | ("HL7" OR "FHIR" OR "CDA") AND ("semantic interoperability" OR "health information exchange") AND ("electronic health records" OR "EHR") |
| HL7 International Repository | ("HL7 v2" OR "HL7 v3" OR "CDA" OR "FHIR") AND ("implementation guide" OR "interoperability standard" OR "semantic model") |
